# The impact of repeated vaccination on relative influenza vaccine effectiveness among vaccinated adults in the United Kingdom

**DOI:** 10.1017/S0950268822001753

**Published:** 2022-11-04

**Authors:** Wey Wen Lim, Benjamin J. Cowling, Georgina Nakafero, Shuo Feng, Jonathan S. Nguyen-Van-Tam, Hikaru Bolt

**Affiliations:** 1 WHO Collaborating Centre for Infectious Disease Epidemiology and Control, School of Public Health, LKS Faculty of Medicine, The University of Hong Kong, Hong Kong Special Administrative Region, China; 2 Laboratory of Data Discovery for Health Limited, Hong Kong Science and Technology Park, New Territories, Hong Kong Special Administrative Region, China; 3 Academic Rheumatology, Faculty of Medicine and Health Sciences, University of Nottingham, Nottingham, UK; 4 Oxford Vaccine Group, University of Oxford, Oxford, UK; 5 Division of Epidemiology and Public Health, School of Clinical Sciences, University of Nottingham, Nottingham, UK; 6 South East and London Field Services, National Infection Service, Public Health England, London, UK; 7 Department of Infectious Disease Epidemiology, Faculty of Epidemiology and Population Health, London School of Hygiene and Tropical Medicine, London, UK

**Keywords:** ‘Influenza vaccines’ [MeSH], ‘Influenza, Human' [MeSH], ‘vaccination’ [MeSH], Clinical Practice Research Datalink, vaccine effectiveness

## Abstract

Annual seasonal influenza vaccination is recommended for individuals at high risk of developing post-infection complications in many locations. However, reduced vaccine immunogenicity and effectiveness have been observed among repeat vaccinees in some influenza seasons. We investigated the impact of repeated influenza vaccination on relative vaccine effectiveness (VE) among individuals who were recommended for influenza vaccination in the United Kingdom with a retrospective cohort study using primary healthcare data from the Clinical Practice Research Datalink, a primary care database in the United Kingdom. Relative VE was estimated against general practitioner-diagnosed influenza-like illnesses (GP-ILI) and medically attended acute respiratory illnesses (MAARI) among participants who have been repeatedly vaccinated compared with first-time vaccinees using proportional hazards models. Relative VE against MAARI may be reduced for individuals above 65 years old who were vaccinated in the current and previous influenza seasons for some influenza seasons. However, these findings were not conclusive as we could not exclude the possibility of residual confounding in our dataset. The use of routinely collected data from electronic health records to examine the effects of repeated vaccination needs to be complemented with sufficient efforts to include negative control outcomes to rule out residual confounding.

## Introduction

Annual influenza vaccination with regularly updated vaccines has been recommended since the 1960s to address frequent antigenic changes in circulating influenza viruses [1, 2]. Concerns about the potential drawbacks of repeated vaccination with influenza vaccines were raised in the 1970s when a small study in a British boarding school reported that children who were vaccinated in previous influenza seasons appeared to have a higher risk of infection compared with first-time vaccinees [3]. While more recent reanalyses of these data identified several flaws in the original study design, analysis and interpretation [1, 4], randomised-controlled trials and cohort studies in the 1980s up to the early 2000s that investigated the impact of repeated vaccination on immunogenicity and clinical effectiveness produced mixed results [5, 6].

More recently, test-negative studies have been increasingly used to estimate influenza vaccine effectiveness (VE) and study the impact of repeated vaccination [7]. Recent attempts to summarise these findings suggested that the effects of repeated vaccination on vaccine immunogenicity and effectiveness can be heterogeneous, possibly due to differences in study populations, influenza seasons and varying effects of antigenic similarity between circulating and vaccine strains [1, 6–10]. Some observations, however, point to some reduction in immunogenicity and VE in repeat vaccinees compared to first-time vaccinees, particularly when the predominant circulating strain is of the influenza A(H3N2) subtype and when there is an antigenic mismatch between the circulating strain and the repeated vaccine strain [1, 11–13]. There is also ecological evidence that shows vaccination programmes which were established earlier and covered more repeat vaccinees yielded lower VE estimates than programmes that were established later [10].

Although vaccination in the current season is still generally found to be more protective compared with no vaccination irrespective of individual vaccination history [1, 7], it is important to understand the impact of repeated vaccination on VE, especially among those indicated for annual vaccination. While existing studies may be limited by the number of influenza seasons studied or by sample size, here we assess the impact of repeat vaccination using the Clinical Practice Research Datalink (CPRD) database, a large primary care database in the United Kingdom with anonymised medical records from approximately 6.9% of the UK population [14]. We compare the incidence of general practitioner-diagnosed influenza-like illness (GP-ILI) and medically attended acute respiratory illnesses (MAARIs) in first-time vaccinees *vs.* repeat vaccinees.

## Methods

### Overview

We conducted a retrospective cohort study to investigate the impact of repeated influenza vaccination on influenza VE in individuals who were repeatedly vaccinated relative to first-time vaccinees in the 2011/12 to 2015/16 seasons using the CPRD database [14]. Demographic and health information from the CPRD GOLD dataset were linked to participating general practitioners' practice level Index of Multiple Deprivation (IMD) and a pregnancy register to provide information on the socioeconomic status and pregnancies of individuals included in this study. The types of vaccines used in the United Kingdom during the study period were egg-grown inactivated or subunit trivalent influenza vaccines. Quadrivalent influenza vaccines were made available from the 2014/15 winter influenza season. Missing data were minimal in this dataset as we were able to extract complete sets of age and IMD information for individuals included in all five seasons. As a high proportion of influenza vaccination in the UK occurs through general practitioners or is prescribed by general practitioners to be completed at pharmacies, we believe the CPRD dataset can capture most vaccination records in their cohort. Hence, the absence of records for vaccination or outcome records was assumed as an absence of vaccination or outcome.

Hazard ratios (HRs) among individuals who received one or more influenza vaccines (identified by medical codes Table S1) over the five influenza seasons preceding each influenza season were estimated against two primary outcomes: (1) GP-ILI and (2) MAARI, which were identified in the CPRD database using medical codes (Table S2). We also estimated the HR for the two primary outcomes for negative control periods or periods outside the influenza season (Table S5) and two negative control outcomes (1) hip fractures and (2) urinary tract infections to investigate the impact of residual confounding on our estimates.

Relative VE is calculated for individuals grouped in two ways: those who were (1) current season vaccinated only *vs.* current season and prior-season vaccinated, and those who were (2) current season vaccinated only *vs.* current season and any vaccination history in the preceding five influenza seasons. This analysis is repeated for negative control outcomes (hip fractures and urinary tract infections) identified during each of the five influenza seasons investigated in this study. The medical codes used to identify hip fractures and urinary tract infections are listed in Tables S3 and S4.

### Study cohorts

For each winter season analysed, age was defined as the age on 1 September immediately before that winter. The age of an individual included in the analysis for the 2015/16 influenza season is their age at 1 September 2015. Individuals aged 65 years or above and those aged between 12 and 64 years in clinical risk groups [15] who had at least one influenza vaccination record (Table S1) for five influenza seasons between 1 January 2010, and 31 December 2016, were ascertained from the CPRD database. As we were interested in the potential impacts of repeated vaccination in those who were indicated for annual influenza vaccination in the United Kingdom, we only extracted data from individuals who received vaccination for any of the five winter seasons during the study period for analysis. We constructed a cohort of vaccinated individuals each year (Table S5) and compared incident events among those who received vaccination in the five preceding years with those who did not to address the question of repeated vaccination effects. Individuals in clinical risk groups were defined as those who were diagnosed with one or more conditions that contribute to the Charlson Comorbidity Index [16]. Individuals were included in final analyses if they were vaccinated between September 1 and the start of each influenza season or negative control period, which was defined as between 30 and 90 calendar days after the end of each influenza season (Table S5). Those who experienced an outcome within 14 days after receipt of influenza vaccination were excluded. Women who were pregnant during this period for each season were also excluded as they were less likely to be indicated for influenza vaccination in the preceding five influenza seasons.

### Statistical analysis

We examined the relationship between the time between the start of each influenza season and the first GP-ILI or MAARI during the influenza season and influenza vaccination status (vaccinated in the current season only or both the current and prior season) with a Cox proportional hazard model on a calendar time axis adjusted for sex, IMD and Charlson Comorbidity Index [17, 18]. Participants who had been vaccinated before the start of each influenza season entered the at-risk period for analysis at the start of each influenza season and exited the at-risk period at the end of the influenza season. If vaccination occurred within 14 days of the start of the influenza season or during the season, participants entered the at-risk period 14 days after influenza vaccination. Participants exited the at-risk period early when death, loss to follow-up due to the individual transferring out of the database, or the outcome of interest occurred.

We also examined the relationship between the time between influenza vaccination and the first GP-ILI and MAARI during each influenza season and the number of influenza vaccinations received in the five preceding influenza seasons using the same Cox proportional hazard model.

We performed stratified analysis for all the analyses described above in individuals <65 years and ≥65 years old as we have only included those who have comorbidities in our analysis for the <65-year-old age group, consistent with UK vaccination policy which targets people ⩾65 years of age irrespective of comorbidities [19, 20]. All analyses were repeated for negative control periods and negative control outcomes. Relative VE for all proportional hazard analyses was calculated as 100 times one minus the adjusted HR (100 × (1 – HR_adj_)%), and 95% confidence intervals were estimated using bootstrapping with 1000 resamples. All analyses were performed using R version 4.1.0 (R Foundation for Statistical Computing, Vienna, Austria) with the ‘survival’ package.

### Patient and public involvement

This study was performed without public involvement. Electronic health records that were routinely collected were analysed and reported without direct contact with participants.

### Ethical approval

The use of CPRD data for secondary analysis in this study was approved via the CPRD's research data governance process, and a separate ethics approval was not required.

### Role of funding source

The funding body for this study did not participate in the design, implementation, analysis and interpretation of this study.

## Results

### Cohort description and outcomes

In the five influenza seasons after the 2009 A(H1N1) pandemic, individuals who received influenza vaccination recorded in the CPRD database each year between 1 September and 31 August the next year decreased from 918 977 in 2011/12 to 536 834 in 2015/16 (Fig. S1). Of these, the records of between 822 541 and 476 616 individuals were included in our analyses after applying the exclusion criteria (Fig. S1). The most common reason for exclusion was the transfer of individuals out of the CPRD dataset before the start of the influenza season (Fig. S1).

The median ages of the vaccinated cohorts in the five influenza seasons were between 74 and 77 years before stratification (Table S6–S10). Most individuals in the vaccinated cohorts (82%–85%) were 65 years old or above (Tables S6–S10). A majority of those above 65 years of age (59%–68%) were vaccinated for all five years preceding each season, compared with 26%–40% of those below 65 years old (Table 1).

**Table 1.** Characteristics of individuals included in the analyses of main outcomes and negative control outcomes for influenza seasons 2011/12 to 2015/16

Description	Season 2011/12		Season 2012/13		Season 2013/14		Season 2014/15		Season 2015/16	
	<65 years	≥65 years	<65 years	≥65 years	<65 years	≥65 years	<65 years	≥65 years	<65 years	≥65 years

Number of individuals included (% total)	127 705 (15.5)	694 836 (84.5)	130 302 (16.4)	662 780 (83.6)	123 074 (17.2)	592 971 (82.8)	110 835 (17.9)	508 514 (82.1)	87 280 (18.3)	389 336 (81.7)
Age (median, s.d.)	53 (13.5)	79 (8.6)	53 (13.4)	79 (8.4)	54 (13.3)	78 (8.2)	54 (13.2)	77 (8.0)	54 (12.9)	76 (7.8)
Male (*n*, % within age group)	60 782 (47.6)	315 663 (45.4)	61 959 (47.6)	302 621 (45.7)	58 471 (47.5)	271 412 (45.8)	52 075 (47.0)	233 261 (45.9)	40 816 (46.8)	178 789 (45.9)
Number of vaccinations received in the past 5 years (*n*, %)										
• 0	17 607 (13.8)	44 151 (6.4)	15 950 (12.2)	34 366 (5.2)	13 845 (11.2)	28 519 (4.8)	11 270 (10.2)	22 026 (4.3)	8486 (9.7)	15 135 (3.9)
• 1	20 092 (15.7)	45 755 (6.6)	17 606 (13.5)	42 409 (6.4)	14 919 (12.1)	32 607 (5.5)	12 452 (11.2)	25 625 (5.0)	9080 (10.4)	17 454 (4.5)
• 2	21 925 (17.2)	53 096 (7.6)	18 812 (14.4)	44 616 (6.7)	15 718 (12.8)	39 471 (6.7)	13 053 (11.8)	29 709 (5.8)	9831 (11.3)	20 887 (5.4)
• 3	17 284 (13.5)	57 283 (8.2)	20 975 (16.1)	55 830 (8.4)	17 405 (14.1)	44 566 (7.5)	14 211 (12.8)	38 351 (7.5)	10 767 (12.3)	26 138 (6.7)
• 4	17 734 (13.9)	83 818 (12.1)	19 732 (15.1)	82 579 (12.5)	21 876 (17.8)	74 450 (12.6)	18 165 (16.4)	59 693 (11.7)	14 219 (16.3)	46 548 (12.0)
• 5	33 063 (25.9)	410 733 (59.1)	37 227 (28.6)	402 980 (60.8)	39 311 (31.9)	373 358 (63.0)	41 684 (37.6)	333 110 (65.5)	34 897 (40.0)	263 174 (67.6)
Vaccinated in season/year (*n*, %)										
• 2010/11	93 972 (73.6)	612 379 (88.1)	98 654 (75.7)	598 986 (90.4)	94 454 (76.7)	537 467 (90.6)	86 460 (78.0)	464 775 (91.4)	69 085 (79.2)	358 475 (92.1)
• 2009/10	88 622 (69.4)	585 056 (84.2)	87 186 (66.9)	558 350 (84.2)	85 207 (69.2)	512 785 (86.5)	78 573 (70.9)	443 872 (87.3)	63 920 (73.2)	345 090 (88.6)
• 2008/09	65 447 (51.2)	542 586 (78.1)	83 445 (64.0)	534 959 (80.7)	75 936 (61.7)	478 072 (80.6)	71 537 (64.5)	423 701 (83.3)	58 386 (66.9)	329 599 (84.7)
• 2007/08	55 409 (43.4)	500 683 (72.1)	61 681 (47.3)	495 602 (74.8)	73 241 (59.5)	458 373 (77.3)	63 885 (57.6)	394 241 (77.5)	53 317 (61.1)	314 223 (80.7)
• 2006/07	48 595 (38.1)	472 029 (67.9)	52 252 (40.1)	456 450 (68.9)	53 791 (43.7)	478 072 (80.6)	61 816 (55.8)	377 829 (74.3)	47 696 (54.6)	292 317 (75.1)
Total number of outcomes (*n*, %)^a^										
• GP-ILI	358 (0.28)	1109 (0.16)	617 (0.5)	1584 (0.2)	202 (0.2)	580 (0.1)	423 (0.4)	1281 (0.3)	301 (0.3)	630 (0.2)
• MAARI	3672 (2.88)	11 587 (1.67)	4679 (3.6)	12 911 (1.9)	2529 (2.1)	6934 (1.2)	3506 (3.2)	9895 (1.9)	2211 (2.5)	5596 (1.4)
Hip fractures	13 (0.01)	897 (0.13)	18 (0.01)	1103 (0.17)	14 (0.01)	654 (0.11)	15 (0.01)	777 (0.15)	14 (0.02)	544 (0.14)
Urinary tract infections	686 (0.54)	7525 (1.08)	899 (0.69)	8226 (1.24)	639 (0.5)	5272 (0.9)	673 (0.6)	5400 (1.06)	524 (0.6)	3862 (0.0)
Charlson Comorbidity Index (*n*, %)										
• 0	0 (0.0)	293 518 (42.2)	0 (0.0)	291 316 (44.0)	0 (0.0)	265 367 (44.8)	0 (0.0)	231 534 (45.5)	0 (0.0)	180 176 (46.3)
• 1–2	108 434 (84.9)	265 755 (38.2)	110 521 (84.8)	254 244 (38.4)	103 636 (84.2)	226 527 (38.2)	92 690 (83.6)	192 146 (37.8)	72 082 (82.6)	144 892 (37.2)
• 3–4	17 138 (13.4)	105 607 (15.2)	17 747 (13.6)	92 829 (14.0)	17 495 (14.2)	80 433 (13.6)	16 310 (14.7)	67 570 (13.3)	13 705 (15.7)	51 441 (13.2)
• >5	2133 (1.7)	29 956 (4.3)	2034 (1.6)	24 391 (3.7)	1943 (1.6)	20 644 (3.5)	1835 (1.7)	17 264 (3.4)	1493 (1.7)	12 827 (3.3)
IMD (n, %)										
• 1–2	41 415 (32.4)	255 726 (36.8)	41 678 (32.0)	241 066 (36.4)	40 385 (32.8)	217 967 (36.8)	36 562 (33.0)	187 676 (36.9)	27 906 (32.0)	141 265 (36.3)
• 3–4	52 323 (41.0)	285 673 (41.1)	53 784 (41.3)	273 722 (41.3)	50 984 (41.4)	248 127 (41.8)	45 701 (41.2)	211 340 (41.6)	35 898 (41.1)	158 550 (40.7)
• 5	33 967 (26.6)	153 437 (22.1)	34 840 (26.7)	147 992 (22.3)	31 705 (25.8)	126 877 (21.4)	28 572 (25.8)	109 498 (21.5)	23 476 (26.9)	89 521 (23.0)

*Note*:

1. The main outcomes are general practitioner-diagnosed influenza like illnesses (GP-ILIs) and medically attended acute respiratory illnesses (MAARIs). Hip fractures and urinary tract infections are negative control outcomes.

a Number of outcomes during the influenza season.

**Fig. 1.**
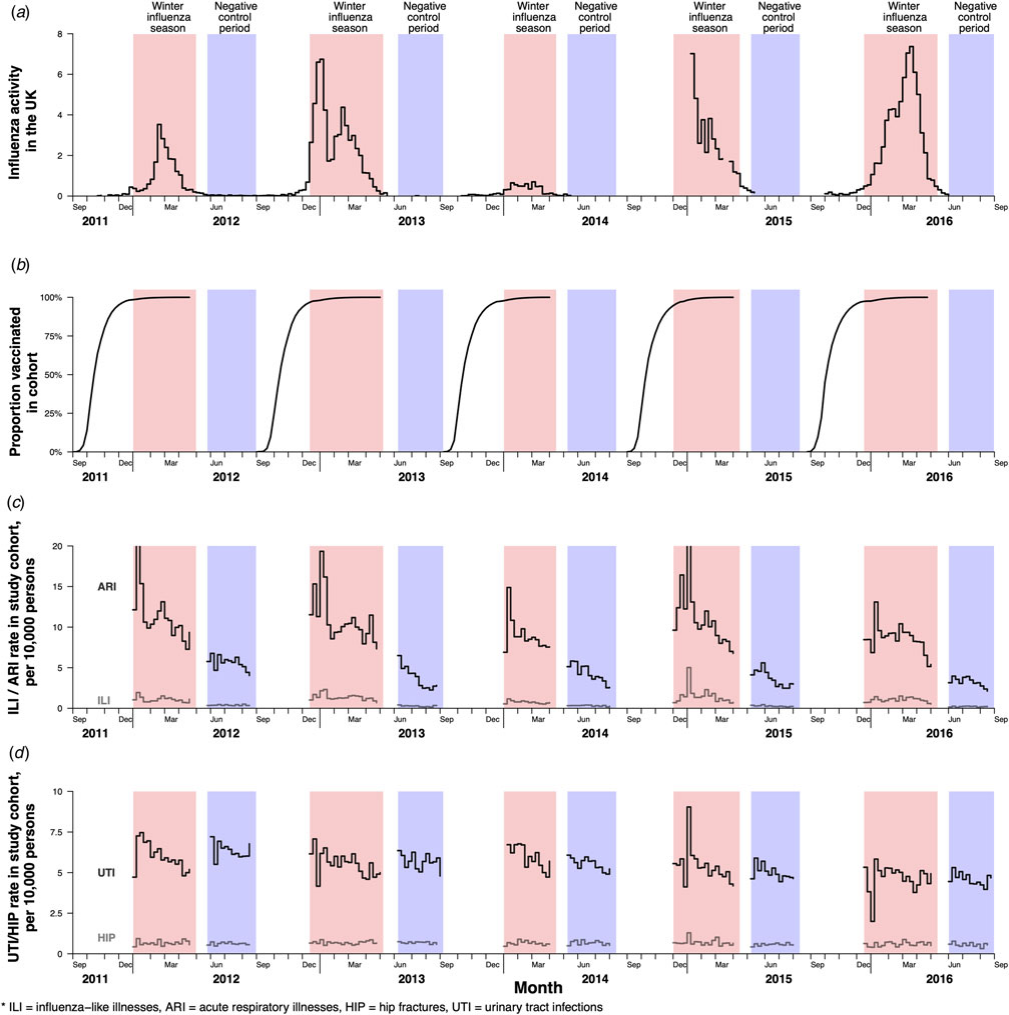
Influenza activity, cumulative vaccination coverage in the study cohorts, and rates of study outcomes in the United Kingdom from September 2011 to September 2016. Influenza seasons are indicated by areas shaded in red, while negative control periods specified in this study are indicated by areas shaded in blue in all panels. (a) Influenza activity in the United Kingdom from the 2011/12 winter influenza season to the 2015/16 influenza season based on national surveillance data. (b) Cumulative proportion of participants vaccinated over time in the study cohort each year. (c) Rates of acute respiratory illnesses and influenza-like illnesses in the study cohorts during the winter influenza seasons and negative control periods each year. (d) Rates of negative control outcomes of hip fractures (HIP) and urinary tract infections (UTI) during the winter influenza seasons and negative control periods each year.

In the United Kingdom, influenza activity peaked each winter, with influenza seasons lasting approximately 15–21 weeks based on national surveillance data (Fig. 1a). The incidence rates of GP-ILI in the CPRD cohorts tracked influenza activity and peaked every winter, while ARI rates that include respiratory illnesses that were related to other pathogens were more spread out throughout the year albeit with winter peaks (Fig. 1c). Most of the vaccinees in our cohorts were vaccinated between October and December (Fig. 1c). The incidence of two negative control outcomes, hip fractures and urinary tract infections, were relatively stable across the winter seasons in all five years (Fig. 1d).

The incidence of GP-ILI and MAARI identified during influenza seasons (Table 1) remained stable in the vaccinated cohorts from 0.1% to 0.2% for GP-ILI and 1% to 2% for MAARI from the 2011/12 to 2015/16 influenza seasons, and higher incidence of both GP-ILI and MAARI was observed in the younger age group for all five seasons (Table 1).

#### Relative VE in first-time vaccinees compared with individuals vaccinated in the current and prior or immediately preceding influenza season

**Fig. 2.**
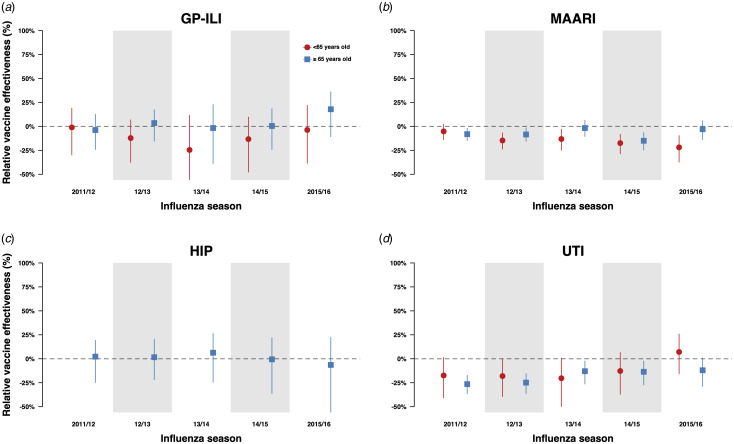
Estimates with 95% confidence intervals of relative VE against GP-ILIs (a), MAARI (b), hip fractures (c) and urinary tract infections (d) during influenza seasons among vaccinees who received influenza vaccination in the previous season compared with first-time vaccinees who have not received any influenza vaccination in the preceding five influenza seasons.

When we compared the incidences of GP-ILI and MAARI among individuals who received influenza vaccinations in the current year only and individuals who received influenza vaccination in both the previous and current year (Fig. 2a), there were no significant differences in relative VE against GP-ILI in both age groups. However, we observed a reduction of relative VE against MAARI in the ≥65-year-old age group in the 2011/12, 2012/13 and 2014/15 influenza seasons, and in the <65-year-old group from the 2012/13 season to the 2015/16 season (Fig. 2b, Table S11).

**Fig. 3.**
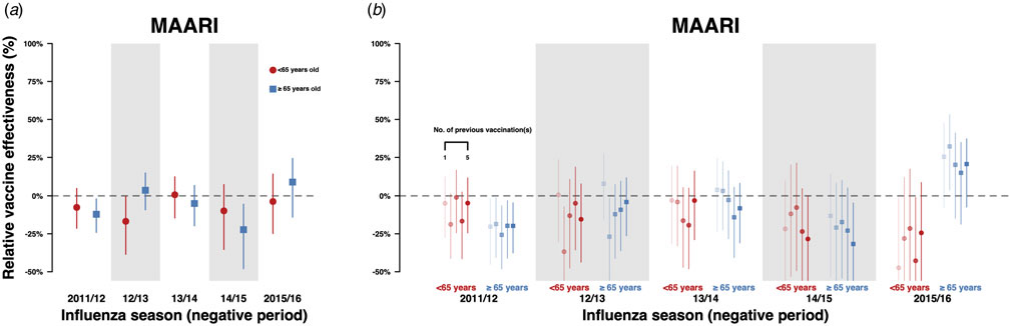
Estimates with 95% confidence intervals of relative VE against MAARI during the negative control period among repeated vaccinees and first-time vaccinees. Relative VE against MAARI among vaccinees who received influenza vaccination in the previous season compared with first-time vaccinees who have not received any influenza vaccination in the previous five influenza seasons is presented in (a). Relative VE against MAARI among vaccinees who received one to five influenza vaccinations (left to right) in the previous five influenza seasons compared with first-time vaccinees who have not received any influenza vaccination in the previous five influenza seasons are presented in (b).

We extracted information on both outcomes in the negative control period for both groups (Figure S2). There were no significant differences in relative VE against MAARI outside influenza seasons between repeatedly vaccinated and first-time vaccinees except for the 2011/12 and 2014/15 seasons in the ≥65-year-old age group (Figs 3a and 3b, Tables S13, S14). The negative control period analysis was not done for GP-ILI due to a low number of cases.

**Fig. 4.**
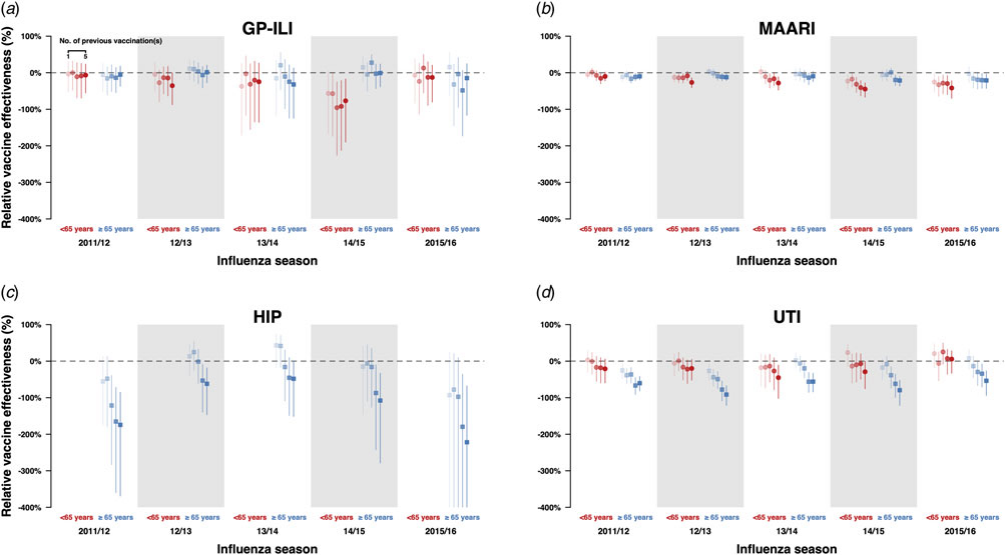
Estimates with 95% confidence intervals of r elative VE against GP-ILIs (a), MAARI (b), hip fractures (c) and urinary tract infections (d) during influenza seasons among vaccinees who received one to five influenza vaccinations (left to right) in the previous five influenza seasons compared with first-time vaccinees who have not received any influenza vaccination in the previous five influenza seasons.

We also conducted the same analyses for negative control outcomes (hip fractures and urinary tract infections) and did not observe significant differences in relative VE against hip fractures in the above 65-year-old age group between those who have been vaccinated in the current year only and those who have also been vaccinated in the previous year (Fig. 2a, Table S15). However, when we compared the incidence of hip fractures among first-time vaccinees and those who were vaccinated in one or more influenza seasons in the previous five influenza seasons, we observed statistically significant reductions of relative VE against these outcomes in the 65-year-old and above group, especially among those who were vaccinated at least three times in the previous 5 influenza seasons (Figs 4c and d, Table S16). Statistically significant reductions of relative VE against urinary tract infections were also observed in repeat vaccinees of the 65-year-old and above age group compared with those who were only vaccinated in the current influenza season (Fig. 2d, Table S15).

#### Relative VE in first-time vaccinees compared with individuals who were vaccinated in the current and one or more of the previous five seasons

When we compared the incidences of GP-ILI and MAARI among those who had also received vaccines in the previous five influenza seasons against first-time vaccinees (Figs 4a and 3b, Table S12), there were no significant differences in relative VE against GP-ILI in individuals who received influenza vaccination in the previous five years for all seasons and age groups except in persons <65 years of age in the 2014/15 season (Fig. 4a, Table S12). Dose-response reductions of relative VE were observed in the same comparison for MAARI, although not all reductions were statistically significant (Fig. 4b, Table S12).

When the incidences of both outcomes identified in the negative control period for these groups were compared, we observed small statistically significant differences only in the 2011/12, 2012/13 and 2015/16 seasons.

In our analyses on negative control outcomes, there was a dose-response reduction of relative VE against hip fractures when those who have received one or more influenza vaccines were compared to those who only received influenza vaccination in the current season, although the reduction was only statistically significant in those who received influenza vaccination for at least three times in the five preceding years (Fig. 4c, Table S12). These analyses were not done in the below 65-year-old age group due to the low number of cases.

Statistically significant reductions of relative VE were observed in the above 65-year-old age group for those who were vaccinated once or more in the preceding five years compared with those who have only been vaccinated in the current season (Fig. 4d, Table S12).

## Discussion

The impact of repeated influenza vaccination on immunogenicity and VE has been a research question of interest for the past five decades [1, 6–10, 21]. In this study, we examined electronic medical records for around 3.4 million person-winters of observation and identified some evidence of small repeat vaccination effects against a non-specific outcome of MAARI (Fig. 2b, Table S11), although not against a more specific outcome of GP-ILI (Fig. 2a, Table S11). However, as there were some statistically significant changes in relative VE against negative control outcomes, we cannot rule out the possibility of residual confounding that may have influenced these observations.

While positive and negative impacts on both outcomes were observed over the years in different locations, recent studies have suggested that these mixed effects could be explained by several phenomena [1, 21]. Cohort effects may result from variation in first exposures to influenza virus infection or vaccination (the original antigenic sin theory) due to anamnestic recall of immune responses to the first influenza strain encountered [1, 22–24]. Another prevailing theory is the antigenic distance hypothesis, proposed by Smith *et al*. [25] According to this hypothesis, negative interference from repeated vaccination could occur when the antigenic distance between vaccine strains for the previous and current season is relatively smaller than the antigenic distance between the currently circulating epidemic strain and the vaccine strain [13, 25].

While this study is not designed to test either of these specific hypotheses, the observed impact of repeated vaccination on relative VE using medical records of vaccinated individuals who were indicated for influenza vaccination in the UK [15] was generally consistent with previous studies, which have found a reduction of point estimates of VE although the results were not statistically significant [5, 11, 26–31]. The observation of VE reduction against GP-ILI and MAARI in the 2014/15 season in repeatedly vaccinated <65-year-olds (Fig. 2d) in particular, could be consistent with the antigenic distance hypothesis, which tends to occur when there is a mismatch between the vaccine and circulating influenza A(H3N2) virus strains [13]. However, for the remaining four influenza seasons, influenza vaccination in the previous year was not associated with a reduction in relative VE against GP-ILI, and neither was an increasing number of influenza vaccinations in the previous five seasons among vaccinated adults above and below the age of 65 years [15].

Notwithstanding, influenza vaccination in the previous season was associated with an increased risk of having MAARI in both groups in the 2012/13 and 2014/15 seasons, as well as in the 2011/12 season in those above 65 years old and in the 2013/14 and 2015/16 season in those below 65 years old. A dose-response reduction in relative VE estimates with an increasing number of vaccinations received in the preceding five influenza seasons was also observed in these seasons (Fig. 4). Coincidentally, the 2011/12, 2012/13 and 2014/15 influenza seasons were seasons in which the influenza A(H3N2) subtype predominated [19, 32–35]. Taken together, these results coincide with the general observation made by Belongia *et al*. in their review that when observed, the negative impacts on VE by repeated vaccination appear to be more pronounced in seasons when the influenza A(H3N2) subtype was the predominant circulating strain [1].

It should be noted that both GP-ILI and MAARI are non-specific endpoints which may include respiratory infections caused by other pathogens. Even though GP-ILI is relatively more specific compared with MAARI, the criteria for such diagnosis are unclear and may vary across general practices. Although the cases of GP-ILI in our study are expected to include relatively fewer non-influenza respiratory illnesses, there is also a possibility that misclassification may still occur as there is currently no formal case definition for GP-ILI that is applied across GP practices in the UK. However, if we assume that the proportion of MAARI caused by influenza in the UK is stable over the five seasons, the reduction in relative VE against MAARI leads us to hypothesise that we might also have observed a reduction in relative VE against laboratory-confirmed influenza if such data had been available.

To assess the potential for residual confounding by factors such as health-seeking behaviour and seasonal effects, we conducted sensitivity analyses by repeating the analyses with MAARI recorded in negative control periods between 30 and 90 calendar days after the end of each influenza season (Fig. 3). Enhancements or reductions of relative VE by repeated vaccination were mostly non-significant in this analysis, indicating that residual confounding after accounting for age by stratification, sex, the Charlson Comorbidity Index and IMD may be reduced. However, as the changes in relative VE by repeated vaccination were statistically significant for some negative control periods and outcomes, we could not rule out the possibility that some residual confounding may still be present. We could only conduct this analysis for MAARI as there were not enough GP-ILI events identified during the negative control periods.

We also conducted sensitivity analyses using hip fractures and urinary tract infections as negative control outcomes [36] as both these outcomes are not related to influenza vaccination or respiratory illnesses. Increases and diminution in relative VE of individuals receiving repeated vaccination were generally not statistically significant. However, some significant reductions of relative VE against hip fractures and urinary tract infections were observed in the above 65-year-old age group. This may indicate the presence of some residual confounding in this age group, for example by frailty or functional status that was not fully reflected by the indicators available in the dataset. Further, first-time vaccinees and repeat vaccinees entering risk periods for influenza virus infections may have different susceptibility and risk profiles, especially at the older end of the age spectrum, where survivorship bias may bias differences in risk away from the null. Attempts to quantify such biases can be informative for the cautious interpretation of repeated vaccination effects in convenient samples from electronic health records [37].

There are a few limitations to our study. We did not access information on individuals in the CPRD database who were not vaccinated in the seasons analysed as we were interested in the potential impacts of repeated vaccination in those who were indicated for annual influenza vaccination in the United Kingdom. Therefore, we limited our analyses to relative VE among vaccinated individuals with different prior vaccination histories and were unable to estimate absolute VE. The use of routinely collected medical data is vulnerable to misclassification of outcomes, particularly when non-specific outcomes for influenza such as GP-ILI and MAARI are used. This may limit our ability to fully capture the effects of repeated vaccinations on VE against influenza infections. The generalisability of these findings is also limited to individuals indicated for vaccination in the UK that uses GPs registered with the CPRD database, precluding investigations into the impact on relative VE against severe influenza and hospitalisation. As we did not access information on influenza vaccination or infection history earlier than the preceding five influenza seasons, which could have permitted additional analyses on overall historical exposure to vaccinations and infections. While hip fractures are unlikely to be related to influenza vaccination and infection, meeting basic requirements for a negative control outcome, differences in health-seeking behaviours following acute respiratory infections and hip fractures may affect its ability to detect confounding effects of similar health-seeking behaviour following acute respiratory infections.

## Conclusions

In conclusion, the findings of this study support general observations in previous studies that any potential evidence of VE reduction by repeated vaccination was insufficient to recommend against annual influenza vaccination, especially in individuals with an elevated risk of influenza and its related complications. Further studies are still important to identify the immunological mechanisms associated with repeat vaccination effects. Large databases of routinely collected electronic medical records are useful resources to support analyses of any repeat vaccination effects, though challenging in disentangling such effects. Nevertheless, diagnostic guidelines and increased laboratory testing for influenza confirmation among medically attended respiratory illnesses would enhance data quality in such datasets.

## Data Availability

The data reported in this paper are available by application directly to the CPRD group.

## Supplementary material

For supplementary material accompanying this paper visit http://doi.org/10.1017/S0950268822001753. click here to view supplementary material
