# Supplementary material for: The impact of repeated vaccination on relative influenza vaccine effectiveness among vaccinated adults in the United Kingdom
Source: Epidemiol Infect. 2022 Nov 4;150:e198. doi: 10.1017/S0950268822001753 (PMC9987024; doi:10.1017/S0950268822001753)
Supplement: Supplementary file 1 [file S0950268822001753sup001.docx]

**Epidemiology and Infection**

**SUPPLEMENTARY MATERIAL**

**THE IMPACT OF REPEATED VACCINATION ON RELATIVE INFLUENZA VACCINE EFFECTIVENESS AMONG VACCINATED ADULTS IN THE UNITED KINGDOM**

Wey Wen Lim^1^*, Benjamin J. Cowling^1,2^, Georgina Nakafero^3^, Shuo Feng^1, 4^, Jonathan S Nguyen-Van-Tam^5^, Hikaru Bolt^6, 7^*

Affiliations:

^1^ WHO Collaborating Centre for Infectious Disease Epidemiology and Control, School of Public Health, Li Ka Shing Faculty of Medicine, The University of Hong Kong, Hong Kong Special Administrative Region, China.

^2^ Laboratory of Data Discovery for Health Limited, Hong Kong Science Park, New Territories, Hong Kong.

^3^ Academic Rheumatology, Faculty of Medicine and Health Sciences, University of Nottingham, Nottingham.

^4^ Oxford Vaccine Group, University of Oxford, Oxford.

^5^ Division of Epidemiology and Public Health, School of Clinical Sciences, University of Nottingham, Nottingham, United Kingdom.

^6^ South East and London Field Services, National Infection Service, Public Health England, London, United Kingdom.

# **Table of contents**

# **Table S1.** Medical/product codes for influenza vaccination 2

# **Table S2.** Medical codes for medically attended acute respiratory illness (MAARI), general-practitioner diagnosed influenza-like illness (ILI), and laboratory-confirmed (LCI) 4

# **Table S3.** Medical codes for hip fractures 6

# **Table S4.** Medical codes for urinary tract infections 8

# **Table S5.** Influenza seasons in the UK from the 2011/12 winter influenza season to the 2015/16 winter influenza season 10

# **Figure S1.** Inclusion and exclusion criteria for individuals included in the main analyses of relative vaccine effectiveness between first-time vaccinees and those who have previously received influenza vaccination 11

# **Figure S2.** Inclusion and exclusion criteria for individuals included in the negative control analyses of relative vaccine effectiveness between first-time vaccinees and those who have previously received influenza vaccination 12

# **Table S6.** Characteristics of individuals included in the study for the 2011/12 winter influenza season 13

# **Table S7.** Characteristics of individuals included in the study for the 2012/13 winter influenza season 14

# **Table S8.** Characteristics of individuals included in the study for the 2013/14 winter influenza season 15

# **Table S9.** Characteristics of individuals included in the study for the 2014/15 winter influenza season 16

# **Table S10.** Characteristics of individuals included in the study for the 2015/16 winter influenza season 17

# **Table S11.** Relative influenza vaccine effectiveness of seasonal influenza vaccines against general practitioner-diagnosed influenza-like illness (GP-ILI) and medically attended acute respiratory illness (MAARI) during influenza seasons among individuals vaccinated in both the current and prior influenza season. 18

# **Table S12**. Relative influenza vaccine effectiveness of seasonal influenza vaccines against general practitioner-diagnosed influenza-like illness (GP-ILI) and medically attended acute respiratory illness (MAARI) during influenza seasons by the number of influenza vaccinations received in the previous 5 seasons 19

# **Table S13.** Relative influenza vaccine effectiveness of seasonal influenza vaccines against medically attended acute respiratory illness (MAARI) outside influenza seasons among individuals vaccinated in both the current and prior influenza season 20

# **Table S14**. Relative influenza vaccine effectiveness of seasonal influenza vaccines against medically attended acute respiratory illness (MAARI) outside influenza seasons by the number of influenza vaccinations received in the previous 5 seasons 21

# **Table S15.** Relative influenza vaccine effectiveness of seasonal influenza vaccines against hip fractures and urinary tract infections during influenza seasons among individuals vaccinated in both the current and prior influenza season 22

# **Table S16.** Relative influenza vaccine effectiveness of seasonal influenza vaccines against hip fractures and urinary tract infections during influenza seasons by the number of influenza vaccinations received in the previous 5 seasons 23

**Table S1.** Medical and product codes for influenza vaccination

| **Medical code** | **Description** |
| --- | --- |
| 6 | Influenza vaccination |
| 9039 | Influenza vacc. administratn. |
| 12104 | Flu vaccination administration |
| 12105 | Influenza immunisation - enhanced services administration |
| 12105 | Influenza immunisation - enhanced services administration |
| 12336 | [V]Influenza vaccination |
| 13025 | [X]Influenza vaccine causing adverse effects therapeutic use |
| 18330 | Has 'flu vaccination at home |
| 18684 | Has 'flu vaccination at hosp. |
| 21123 | [V]Flu - influenza vaccination |
| 32942 | Has influenza vaccination at work |
| 35655 | Influenza vacc.administrat.NOS |
| 44555 | Has'flu vaccination at surgery |
| 94301 | First pandemic influenza vaccination |
| 95092 | Second pandemic influenza vaccination |
| 97941 | Influenza vaccination given by other healthcare provider |
| 98183 | PANDEMRIX - first influenza A (H1N1v) 2009 vaccination given |
| 98184 | PANDEMRIX - second influenza A (H1N1v) 2009 vaccination give |
| 98203 | PANDEMRIX - 1st flu A (H1N1v) 2009 vac by othr hlth provider |
| 98217 | 1st pandemic influenza vacc give by other healthcare providr |
| 98234 | CELVAPAN - first influenza A (H1N1v) 2009 vaccination given |
| 98302 | CELVAPAN - second influenza A (H1N1v) 2009 vaccination given |
| 98303 | CELVAPAN - 2nd flu A (H1N1v) 2009 vacc by othr hlth provider |
| 98304 | PANDEMRIX - 2nd flu A (H1N1v) 2009 vac by othr hlth provider |
| 98306 | 2nd pandemic influenza vacc give by other healthcare providr |
| 98449 | CELVAPAN - 1st flu A (H1N1v) 2009 vacc by othr hlth provider |
| 104688 | Seasonal influenza vaccination |
| 105077 | Seasonal influenza vaccin given by other healthcare provider |
| 105195 | Seasonal influenza vaccination given by pharmacist |
| 106994 | Administration of first intranasal influenza vaccination |
| 106995 | Administration of second intranasal influenza vaccination |
| 107156 | Administration of intranasal influenza vaccination |
| 107297 | Administration of first intranasal seasonal influenza vacc |
| 107315 | Administration of first intranasal pandemic influenza vacc |
| 107352 | Administration of second intranasal seasonal influenza vacc |
| 107413 | First intranasal seasonal flu vacc gvn by othr hlthcare prov |
| 107573 | Seasonal influenza vaccination given while hospital inpt |
| 107646 | Administration of second intranasal pandemic influenza vacc |
| 107730 | Secnd intranasal seasonal flu vacc gvn by othr hlthcare prov |
| 108772 | First intranasal pndmc influenza vcc gvn othr hlthcare prvdr |
| 110182 | 1st intramuscular seasonal influenza vacc given by other HCP |
| 110219 | 2nd intramuscular seasonal influenza vacc given by other HCP |
| 110854 | Administration of first inactivated seasonal influenza vacc |

| **Product code** | **Description** |
| --- | --- |
| 398 | Influenza inactivated split virion Vaccination (Aventis Pasteur MSD) |
| 639 | Influenza vaccine (split virion, inactivated) suspension for injection 0.5ml pre-filled syringes |
| 834 | Begrivac vaccine suspension for injection 0.5ml pre-filled syringes (Novartis Vaccines and Diagnostics Ltd) |
| 922 | Influenza inactivated surface antigen Vaccination |
| 1329 | Fluvirin vaccine suspension for injection 0.5ml pre-filled syringes (Novartis Vaccines and Diagnostics Ltd) |
|  | *(Continued on the next page…)* |
|  |  |
|  |  |
| **Product code** | **Description** |
| *(… continued from the previous page)* | |
| 2139 | Fluarix vaccine suspension for injection 0.5ml pre-filled syringes (GlaxoSmithKline UK Ltd) |
| 2552 | Influvac Sub-unit vaccine suspension for injection 0.5ml pre-filled syringes (BGP Products Ltd) |
| 2601 | Mfv-ject Vaccination (Aventis Pasteur MSD) |
| 9710 | Agrippal vaccine suspension for injection 0.5ml pre-filled syringes (Seqirus Vaccines Ltd) |
| 10030 | Inflexal V vaccine suspension for injection 0.5ml pre-filled syringes (Janssen-Cilag Ltd) |
| 11824 | Enzira vaccine suspension for injection 0.5ml pre-filled syringes (Pfizer Ltd) |
| 13595 | Fluzone Vaccination (Aventis Pasteur MSD) |
| 16585 | Viroflu vaccine suspension for injection 0.5ml pre-filled syringes (Janssen-Cilag Ltd) |
| 18612 | Mastaflu vaccine suspension for injection 0.5ml pre-filled syringes (Masta Ltd) |
| 19684 | INFLUVAC DISPOSABLE SYRINGE |
| 24779 | Influenza inactivated split virion Paediatric vaccination |
| 27407 | Imuvac vaccine suspension for injection 0.5ml pre-filled syringes (BGP Products Ltd) |
| 30156 | Invivac vaccine suspension for injection 0.5ml pre-filled syringes (Abbott Healthcare Products Ltd) |
| 30198 | Influenza inactivated split virion Vaccination (sanofi pasteur MSD Ltd) |
| 32391 | Influenza vaccine (surface antigen, inactivated) suspension for injection 0.5ml pre-filled syringes (Novartis Vaccines and Diagnostics Ltd) |
| 38421 | Influenza inactivated split virion Vaccination (Evans Vaccines Ltd) |
| 40760 | Influenza vaccine (split virion, inactivated) 15microgram strain suspension for injection 0.1ml pre-filled syringes |
| 40876 | Influenza vaccine (split virion, inactivated) 9microgram strain suspension for injection 0.1ml pre-filled syringes |
| 41150 | Pandemrix vaccine emulsion and suspension for emulsion for injection (GlaxoSmithKline UK Ltd) |
| 41168 | Influenza H1N1 vaccine (split virion, inactivated, adjuvanted) emulsion and suspension for emulsion for injection |
| 41240 | Influenza H1N1 vaccine (whole virion, Vero cell derived, inactivated) suspension for injection |
| 41925 | Celvapan (H1N1) vaccine (whole virion, Vero cell derived, inactivated) suspension for injection (Baxter Healthcare Ltd) |
| 43825 | Intanza 15microgram strain vaccine suspension for injection 0.1ml pre-filled syringes (sanofi pasteur MSD Ltd) |
| 43827 | Intanza 9microgram strain vaccine suspension for injection 0.1ml pre-filled syringes (sanofi pasteur MSD Ltd) |
| 44759 | INFLUENZA PRE-FILLED SYRINGE |
| 45661 | Influenza vaccine (split virion, inactivated) suspension for injection 0.5ml pre-filled syringes (Pfizer Ltd) |
| 47932 | Fluenz vaccine nasal suspension 0.2ml unit dose (AstraZeneca UK Ltd) |
| 48085 | Influenza inactivated split virion Vaccination (Chiron UK Ltd) |
| 48658 | Influenza vaccine (split virion, inactivated) suspension for injection 0.5ml pre-filled syringes (sanofi pasteur MSD Ltd) |
| 48740 | Influenza vaccine (surface antigen, inactivated) suspension for injection 0.5ml pre-filled syringes |
| 49716 | Influenza vaccine (surface antigen, inactivated, virosome) suspension for injection 0.5ml pre-filled syringes |
| 51087 | Optaflu vaccine suspension for injection 0.5ml pre-filled syringes (Seqirus Vaccines Ltd) |
| 51289 | Influenza vaccine (live attenuated) nasal suspension 0.2ml unit dose |
| 54677 | Preflucel vaccine suspension for injection 0.5ml pre-filled syringes (Baxter Healthcare Ltd) |
| 57140 | Influenza vaccine (live attenuated) nasal suspension 0.2ml unit dose |
| 57401 | Influvac Desu vaccine suspension for injection 0.5ml pre-filled syringes (Abbott Healthcare Products Ltd) |
| 57678 | Fluenz vaccine nasal suspension 0.2ml unit dose (AstraZeneca UK Ltd) |
| 57917 | Fluarix Tetra vaccine suspension for injection 0.5ml pre-filled syringes (GlaxoSmithKline UK Ltd) |
| 61580 | Influenza vaccine (split virion, inactivated) suspension for injection 0.25ml pre-filled syringes |
| 61792 | Fluenz Tetra vaccine nasal suspension 0.2ml unit dose (AstraZeneca UK Ltd) |
| 61898 | Influenza vaccine (split virion, inactivated) suspension for injection 0.5ml pre-filled syringes (A A H Pharmaceuticals Ltd) |
| 63690 | Inflexal V suspension for injection 0.5ml pre-filled syringes (sanofi pasteur MSD Ltd) |
| 65205 | FluMist Quadrivalent vaccine nasal suspension 0.2ml unit dose (AstraZeneca UK Ltd) |

# **Table S2.** Medical codes for medically attended acute respiratory illness (MAARI), general-practitioner diagnosed influenza-like illness (ILI), and laboratory-confirmed (LCI)

| **Medical codes** | **Description** | **MAARI** | **ILI** | **LCI** |
| --- | --- | --- | --- | --- |
| 76 | Upper respiratory infection NOS | X |  |  |
| 556 | Influenza | X | X |  |
| 2157 | Flu like illness | X | X |  |
| 2637 | Upper respiratory tract infection NOS | X |  |  |
| 4221 | Recurrent upper respiratory tract infection | X |  |  |
| 5947 | Influenza like illness | X | X |  |
| 6094 | Pneumonia or influenza NOS | X | X |  |
| 6294 | Acute upper respiratory tract infection | X |  |  |
| 6421 | Viral upper respiratory tract infection NOS | X |  |  |
| 8980 | Influenza-like symptoms | X | X |  |
| 10086 | Pneumonia and influenza | X | X |  |
| 11849 | Other specified pneumonia or influenza | X | X |  |
| 13573 | Influenza with bronchopneumonia | X | X |  |
| 14791 | Influenza with gastrointestinal tract involvement | X | X |  |
| 15774 | Influenza with laryngitis | X | X |  |
| 15912 | Influenza with pneumonia | X | X |  |
| 16116 | Other upper respiratory tract disease NOS | X |  |  |
| 16388 | Influenza NOS | X | X |  |
| 23488 | Influenza with respiratory manifestations NOS | X | X |  |
| 26010 | Other acute upper respiratory infections | X |  |  |
| 27072 | Influenza A antibody level | X | X | X |
| 27073 | Influenza B antibody level | X | X | X |
| 29457 | Chest infection - influenza with pneumonia | X | X |  |
| 29466 | Upper respiratory tract disease NOS | X |  |  |
| 29617 | Influenza with pharyngitis | X | X |  |
| 31363 | Influenza with other manifestations NOS | X | X |  |
| 32802 | Other upper respiratory tract diseases | X |  |  |
| 35745 | Influenza with pneumonia NOS | X | X |  |
| 39041 | Other specified diseases of upper respiratory tract | X |  |  |
| 43625 | Influenza with other respiratory manifestation | X | X |  |
| 45892 | Influenza A antigen level | X | X | X |
| 46157 | Influenza with encephalopathy | X | X |  |
| 47472 | Influenza with other manifestations | X | X |  |
| 48453 | Influenza B antigen level | X | X | X |
| 53055 | [X]Acute upper respiratory infections | X |  |  |
| 55646 | Acute myocarditis - influenza | X | X |  |
| 57308 | Other upper respiratory tract diseases NOS | X |  |  |
| 62632 | Influenza with pneumonia, influenza virus identified | X | X | X |
| 68281 | [X]Other diseases of the upper respiratory tract | X |  |  |
| 69335 | Encephalitis due to influenza-specific virus not identified | X | X |  |
| 70941 | [X]Other specified diseases of upper respiratory tract | X |  |  |
| 71658 | Encephalitis due to influenza-virus identified | X | X | X |
| 92944 | Influenza (A&B) serology | X | X | X |
| 94930 | Avian influenza | X | X |  |
| 96017 | Influenza B virus detected | X | X | X |
| 96018 | Influenza H3 virus detected |  |  |  |
| 96019 | Influenza H1 virus detected | X | X | X |
|  | *(Continued on the next page…)* | | | |

| **Medical codes** | **Description** | **MAARI** | **ILI** | **LCI** |
| --- | --- | --- | --- | --- |
| *(… continued from the previous page)* | | | | |
| 96599 | Amantadine resistant virus detected | X | X | X |
| 97062 | Influenza A virus, other or untyped strain detected | X | X | X |
| 97279 | [X]Influenza+other manifestations, virus not identified | X | X | X |
| 97605 | [X]Influenza+oth respiratory manifestatns,virus not identifd | X | X |  |
| 97936 | [X]Influenza+other manifestations,influenza virus identified | X | X |  |
| 98102 | Influenza A (H1N1) swine flu | X | X | X |
| 98103 | Possible influenza A virus H1N1 subtype | X | X |  |
| 98115 | Suspected swine influenza | X | X |  |
| 98125 | Suspected influenza A virus subtype H1N1 infection | X | X |  |
| 98129 | Influenza due to Influenza A virus subtype H1N1 | X | X |  |
| 98143 | Influenza A virus H1N1 subtype detected | X | X | X |
| 98156 | Influenza H5 virus detected | X | X | X |
| 98257 | [X]Flu+oth respiratory manifestations,'flu virus identified | X | X | X |
| 98331 | Influenza A virus H1N1 subtype not detected | X | X | X |
| 99840 | Oseltamivir resistant virus detected | X | X |  |
| 100014 | Telephone consultation for suspected swine flu | X | X | X |
| 100146 | Telephne consultatn suspected influenza A virus subtype H1N1 | X | X |  |
| 101845 | Influenza A virus subtype H1N1 serology | X | X |  |
| 102918 | Influenza H2 virus detected | X | X | X |
| 104016 | Influenza A nucleic acid detection | X | X | X |
| 105541 | Influenza B nucleic acid detection | X | X | X |
| 109037 | Influenza A virus ribonucleic acid detection assay | X | X | X |
| 109038 | Influenza B virus ribonucleic acid detection assay | X | X | X |
| 109160 | Influenza A nucleic acid detection assay | X | X | X |
| 109161 | Influenza B nucleic acid detection assay | X | X | X |

# **Table S3.** Medical codes for hip fractures

| **Medical code** | **Description** |
| --- | --- |
| 1591 | Closed fracture acetabulum |
| 1994 | Hip fracture |
| 2225 | Fracture of neck of femur |
| 5301 | Closed fracture of proximal femur, pertrochanteric |
| 8243 | Subtrochanteric fracture |
| 8648 | Closed fracture of femur, intertrochanteric |
| 9072 | Fracture of acetabulum |
| 10570 | Hip fracture NOS |
| 17019 | Cls # prox femur, subcapital, Garden grade unspec. |
| 18273 | Closed fracture of neck of femur NOS |
| 19117 | Cls # proximal femur, trochanteric section, unspecified |
| 19387 | Closed fracture of femur, greater trochanter |
| 23803 | Open fracture proximal femur,subcapital, Garden grade III |
| 24276 | Closed fracture of unspecified proximal femur |
| 24587 | Fracture-dislocation or subluxation hip |
| 28375 | Closed fracture of pelvis NOS |
| 28965 | Pertrochanteric fracture |
| 29145 | Closed fracture proximal femur, subtrochanteric |
| 33957 | Closed fracture proximal femur, subcapital, Garden grade II |
| 34078 | Closed fracture proximal femur, subcapital, Garden grade IV |
| 34212 | Closed fracture-subluxation of pelvis |
| 34351 | Closed fracture proximal femur, subcapital, Garden grade I |
| 34910 | Closed fracture-dislocation of pelvis |
| 36391 | Closed fracture head of femur |
| 36599 | Closed fracture proximal femur, subcapital, Garden grade III |
| 38054 | Open fracture of neck of femur NOS |
| 38489 | Closed fracture proximal femur, transcervical |
| 38878 | Open fracture proximal femur,subcapital, Garden grade unspec |
| 39396 | Open fracture of femur, intertrochanteric |
| 39984 | Cls # prox femur, intracapsular section, unspecified |
| 40267 | Closed fracture-dislocation, hip joint |
| 44735 | Cls # of proximal femur, pertrochanteric section, NOS |
| 45141 | Closed fracture proximal femur, intertrochanteric, two part |
| 45527 | Closed fracture acetabulum NOS |
| 45779 | Closed fracture of femur, upper epiphysis |
| 48337 | Closed fracture of femur, lesser trochanter |
| 49209 | Closed fracture proximal femur, other transcervical |
| 50727 | Opn # proximal femur, intracapsular section, unspecified |
| 51216 | Cls # proximal femur, intertrochanteric, comminuted |
| 51861 | Closed fracture, base of neck of femur |
| 51999 | Open fracture proximal femur,subcapital, Garden grade IV |
| 52194 | Closed fracture proximal femur, basicervical |
| 53566 | Open fracture acetabulum |
| 57923 | Closed fracture acetabulum, posterior column |
| 58642 | Open fracture of unspecified proximal femur |
| 58720 | Open fracture-dislocation, hip joint |
| 59904 | Other specified closed fracture acetabulum |
| 60885 | Open fracture proximal femur,subcapital, Garden grade I |
| 61733 | Open fracture of proximal femur, pertrochanteric |
| 62562 | Other specified open fracture acetabulum |
| 62966 | Closed fracture proximal femur, transcervical, NOS |
|  |  |
|  |  |
|  |  |
|  | *(Continued on next page…)* |
| **Medical code** | **Description** |
| *(… continued from previous page)* | |
| 64777 | Open fracture acetabulum NOS |
| 65690 | Closed fracture proximal femur, midcervical section |
| 67394 | Open fracture proximal femur,subcapital, Garden grade II |
| 67633 | Open # of proximal femur, trochanteric section, unspecified |
| 68229 | Closed fracture of femur, subcapital |
| 68668 | Open fracture proximal femur, other transcervical |
| 69418 | Closed fracture acetabulum, floor |
| 69919 | Closed fracture proximal femur, transepiphyseal |
| 70479 | Open fracture of proximal femur, pertrochanteric, NOS |
| 71282 | Open fracture proximal femur, subtrochanteric |
| 72138 | Open fracture proximal femur, transepiphyseal |
| 72525 | Closed fracture acetabulum, anterior lip alone |
| 73210 | Open fracture head, femur |
| 73234 | Open fracture of femur, subcapital |
| 73479 | Closed fracture acetabulum, anterior column |
| 73981 | Open fracture proximal femur, transcervical |
| 93374 | Closed fracture-subluxation, hip joint |
| 94649 | Closed fracture acetabulum, posterior lip alone |
| 96518 | Open fracture of femur, upper epiphysis |
| 96644 | Open fracture of femur, greater trochanter |
| 96984 | Open fracture of ilium, unspecified |
| 97971 | Open fracture proximal femur, intertrochanteric, comminuted |
| 98267 | Closed fracture acetabulum, double column unspecified |
| 100771 | Open fracture base of neck of femur |
| 101567 | Open fracture proximal femur, intertrochanteric, two part |
| 112712 | Open fracture proximal femur, transcervical, NOS |

# **Table S4.** Medical codes for urinary tract infections

| **Medical code** | **Description** |
| --- | --- |
| 150 | Urinary tract infection, site not specified NOS |
| 1289 | Urinary tract infection, site not specified |
| 1805 | Infections of kidney |
| 1899 | Pyelonephritis unspecified |
| 2546 | Acute pyelonephritis |
| 2939 | Calculous pyelonephritis |
| 3610 | Perinephric abscess |
| 4255 | Infection of kidney NOS |
| 4453 | Pyuria, site not specified |
| 4993 | bacteriuria, site not specified |
| 5721 | Urinary tract infection following delivery |
| 10233 | Asymptomatic bacteriuria |
| 12570 | Post-operative urinary tract infection |
| 14711 | Pyonephrosis unspecified |
| 14828 | Acute pyelitis |
| 15357 | Renal abscess |
| 21017 | Renal infections |
| 23772 | Renal and perinephric abscess |
| 33296 | Urethral swab culture positive |
| 34347 | Urethral abscess |
| 34645 | Abscess of bladder |
| 37384 | Infect+inflam react due pros dev, implt+graft in urinary syst |
| 38698 | Acute pyelonephritis NOS |
| 47249 | Urethral and periurethral abscess |
| 49212 | Renal carbuncle |
| 53944 | Pyelonephritis And Pyonephrosis Unspecified |
| 59121 | Unspecified Pyelonephritis Nos |
| 64482 | Acute pyelonephritis without medullary necrosis |
| 65773 | Infestation of renal pelvis with ureter |
| 71871 | Urethral abscess NOS |
| 73412 | Renal and perinephric abscess NOS |
| 95710 | Pyelonephritis In Diseases Ec |
| 96410 | Urethral stricture due to unspecified infection |
| 96671 | Urethral stricture due to infection EC |
| 97002 | Urinary tract infection |
| 100354 | Urethral abscess unspecified |
| 101196 | Infective urethral stricture NOS |
| 107568 | Catheter-associated urinary tract infection |
| 107843 | CAUTI - catheter-associated urinary tract infection |
| 110092 | Emphysematous pyelonephritis |

# **Table S5.** Influenza seasons in the UK from the 2011/12 winter influenza season to the 2015/16 winter influenza season

| **Season^†^** | **Influenza positivity threshold** | **Influenza season** | | | **Negative control period^#^** | |  |  |  |
| --- | --- | --- | --- | --- | --- | --- | --- | --- | --- |
|  |  | **Start** | **End** | **Duration (weeks)** | **Start** | **End** | **Peak GP ILI consultations^ (week/year)** | **Circulating strain** | **Antigenic similarity of circulating and vaccine strain** |
| 2011/12 | 5.0% | Dec 26, 2011 | Apr 22, 2012 | 17 | May 22, 2012 | Aug 20, 2012 | 7/2012 | A(H3) | Similar, some antigenic drift in A(H3) strains |
| 2012/13 | 5.0% | Dec 10, 2012 | May 4, 2013 | 23 | Jun 3, 2013 | Aug 31, 2013 | 52/2012 | B, A(H3) | Similar |
| 2013/14 | 5.0% | Dec 30, 2013 | Apr 6, 2014 | 14 | May 6, 2014 | Aug 4, 2014 | 3/2014 | A(H1N1)pdm09, A(H3N2) | Similar |
| 2014/15 | 5.0%* | Dec 01, 2014 | Apr 12, 2015 | 19 | May 12, 2015 | Aug 10, 2015 | 52/2014 | A(H3N2), B | Majority similar  23% circulating strains drifted |
| 2015/16 | 7.4%* | Dec 21, 2015 | May 1, 2016 | 19 | May 31, 2016 | Aug 29, 2016 | 11/2016 | A(H1N1)pdm09,  B | Similar |

^†^ Information on influenza seasons, general practitioner consultations for influenza-like illnesses, circulating strains, and antigenic similarity of circulating and vaccine strains were sourced from annual reports by the Health Protection Agency (2011/12 season) and Public Health England (seasons 2012/13 – 2015/16).

^#^ The negative control period in this study is defined as the period between 30 and 90 calendar days (total = 60 calendar days) after the end of each influenza season.

^ GP = general practitioner, ILI = influenza-like illness

* Threshold estimated by the Moving Epidemic Method, which was used from the 2014/2015 influenza season onwards.

**Note:** The information in this table was identified from the *Surveillance of influenza and other respiratory viruses in the United Kingdom* reports from the 2011/12 winter influenza season to the 2015/16 winter influenza seasons below:

1. Health Protection Agency. Surveillance of influenza and other respiratory pathogens in the UK, 2011/12. [Internet]. 2012 [cited 2020 August 20]. Available from: <https://webarchive.nationalarchives.gov.uk/20140714113118/http:/www.hpa.org.uk/webc/HPAwebFile/HPAweb_C/1317134705939>.

2. Public Health England. Surveillance of influenza and other respiratory viruses, including novel respiratory viruses, in the United Kingdom: Winter 2012/13. [Internet]. 2013 [cited 2020 August 22]. Available from: <https://assets.publishing.service.gov.uk/government/uploads/system/uploads/attachment_data/file/325217/Annual_flu_report_winter_2012_to_2013.pdf>

3. Public Health England. Surveillance of influenza and other respiratory viruses in the United Kingdom: Winter 2013/14. [Internet]. 2014 [cited 2020 August 22]. Available from: <https://assets.publishing.service.gov.uk/government/uploads/system/uploads/attachment_data/file/325203/Flu_annual_report_June_2014.pdf>

4. Public Health England. Surveillance of influenza and other respiratory viruses in the United Kingdom: winter 2014 to 2015. [Internet]. 2015 [cited 2020 August 22]. Available from: <https://assets.publishing.service.gov.uk/government/uploads/system/uploads/attachment_data/file/429617/Annualreport_March2015_ver4.pdf>

5. Public Health England. Surveillance of influenza and other respiratory viruses in the United Kingdom: Winter 2015 to 2016. [Internet]. 2016 [cited 2020 August 22]. Available from: <https://assets.publishing.service.gov.uk/government/uploads/system/uploads/attachment_data/file/526405/Flu_Annual_Report_2015_2016.pdf>

**Figure S1.** Inclusion and exclusion criteria for individuals included in the main analyses of relative vaccine effectiveness between first-time vaccinees and those who have previously received influenza vaccination

**Figure S2.** Inclusion and exclusion criteria for individuals included in the negative control analyses of relative vaccine effectiveness between first-time vaccinees and those who have previously received influenza vaccination

# **Table S6.** Characteristics of individuals included in the study for the 2011/12 winter influenza season

| Characteristics | **Overall** | **< 65 years** | **≥ 65 years** | p-value^1^ |
| --- | --- | --- | --- | --- |
| N (%) | 822,541 | 127,705 (15.5) | 694,836 (84.5) |  |
| Age (median, SD) | 77 (14.9) | 53 (13.5) | 79 (8.6) | < 0.001 |
| Male (n, %) | 376,445 (45.8) | 60,782 (47.6) | 315,663 (45.4) | < 0.001 |
| Number of vaccinations in previous years (n, %)  0  1  2  3  4  5 | 61,758 (7.5)  65,847 (8.0)  75,021 (9.1)  74,567 (9.1)  101,552 (12.3)  443,796 (54.0) | 17,607 (13.8)  20,092 (15.7)  21,925 (17.2)  17,284 (13.5)  17,734 (13.9)  33,063 (25.9) | 44,151 (6.4)  45,755 (6.6)  53,096 (7.6)  57,283 (8.2)  83,818 (12.1)  410,733 (59.1) | < 0.001 |
| Vaccinated in season/year (n, %)  2010/11  2009/10  2008/09  2007/08  2006/07 | 706,351 (85.9)  673,678 (81.9)  608,033 (73.9)  556,092 (67.6)  520,624 (63.3) | 93,972 (73.6)  88,622 (69.4)  65,447 (51.2)  55,409 (43.4)  48,595 (38.1) | 612,379 (88.1)  585,056 (84.2)  542,586 (78.1)  500,683 (72.1)  472,029 (67.9) | < 0.001  < 0.001  < 0.001  < 0.001  < 0.001 |
| Total number of outcomes in year 2011/2012^2^ (n, %)  ILI  ARI | 1,467 (0.17)  15,259 (1.86) | 358 (0.28)  3,672 (2.88) | 1,109 (0.16)  11,587 (1.67) | < 0.001  < 0.001 |
| Hip fractures (n, %) | 910 (0.11) | 13 (0.01) | 897 (0.13) | < 0.001 |
| Urinary tract infections (n, %) | 8,211 (1.00) | 686 (0.54) | 7,525 (1.08) | < 0.001 |
| Charlson Comorbidity Index (n, %)^3^  0  1 - 2  3 - 4  > 5 | 293,518 (35.7)  374,189 (45.5)  122,745 (14.9)  32,089 (3.9) | 0 (0.0)  108,434 (84.9)  17,138 (13.4)  2,133 (1.7) | 293,518 (42.2)  265,755 (38.2)  105,607 (15.2)  29,956 (4.3) | < 0.001 |
| Index of multiple deprivation  1 - 2  3 – 4  5 | 297,141 (36.1)  337,996 (41.1)  187,404 (22.8) | 41,415 (32.4)  52,323 (41.0)  33,967 (26.6) | 255,726 (36.8)  285,673 (41.1)  153,437 (22.1) | < 0.001 |

# **Table S7.** Characteristics of individuals included in the study for the 2012/13 winter influenza season

| Characteristics | **Overall** | **< 65 years** | **≥ 65 years** | p-value^1^ |
| --- | --- | --- | --- | --- |
| N (%) | 793,082 | 130,302 (16.4) | 662,780 (83.6) |  |
| Age (median, SD) | 76 (14.7) | 53 (13.4) | 79 (8.4) | < 0.001 |
| Male (n, %) | 364,580 (46.0) | 61,959 (47.6) | 302,621 (45.7) | < 0.001 |
| Number of vaccinations in previous years (n, %)  0  1  2  3  4  5 | 50,316 (6.3)  60,015 (7.6)  63,428 (8.0)  76,805 (9.7)  102,311 (12.9)  440,207 (55.5) | 15,950 (12.2)  17,606 (13.5)  18,812 (14.4)  20,975 (16.1)  19,732 (15.1)  37,227 (28.6) | 34,366 (5.2)  42,409 (6.4)  44,616 (6.7)  55,830 (8.4)  82,579 (12.5)  402,980 (60.8) | < 0.001 |
| Vaccinated in season/year (n, %)  2011/12  2010/11  2009/10  2008/09  2007/08 | 697,640 (88.0)  645,536 (81.4)  618,404 (78.0)  557,283 (70.3)  508,702 (64.1) | 98,654 (75.7)  87,186 (66.9)  83,445 (64.0)  61,681 (47.3)  52,252 (40.1) | 598,986 (90.4)  558,350 (84.2)  534,959 (80.7)  495,602 (74.8)  456,450 (68.9) | < 0.001  < 0.001  < 0.001  < 0.001  < 0.001 |
| Total number of outcomes in year 2012/2013^2^ (n, %)  ILI  ARI | 2,201 (0.3)  17,590 (2.2) | 617 (0.5)  4,679 (3.6) | 1,584 (0.2)  12,911 (1.9) | < 0.001  < 0.001 |
| Hip fractures (n, %) | 1,121 (0.14) | 18 (0.01) | 1,103 (0.17) | < 0.001 |
| Urinary tract infections (n, %) | 9,125 (1.15) | 899 (0.69) | 8,226 (1.24) | < 0.001 |
| Charlson Comorbidity Index (n, %)^3^  0  1 - 2  3 - 4  > 5 | 291,316 (36.7)  364,765 (46.0)  110,576 (13.9)  26,425 (3.3) | 0 (0.0)  110,521 (84.8)  17,747 (13.6)  2,034 (1.6) | 291,316 (44.0)  254,244 (38.4)  92,829 (14.0)  24,391 (3.7) | < 0.001 |
| Index of multiple deprivation  1 - 2  3 – 4  5 | 282,744 (35.7)  327,506 (41.3)  182,832 (23.1) | 41,678 (32.0)  53,784 (41.3)  34,840 (26.7) | 241,066 (36.4)  273,722 (41.3)  147,992 (22.3) | < 0.001 |

# **Table S8.** Characteristics of individuals included in the study for the 2013/14 winter influenza season

| Characteristics | **Overall** | **< 65 years** | **≥ 65 years** | p-value^1^ |
| --- | --- | --- | --- | --- |
| N | 716,045 | 123,074 (17.2) | 592,971 (82.8) |  |
| Age (median, SD) | 75 (14.5) | 54 (13.3) | 78 (8.2) | < 0.001 |
| Male (n, %) | 329,883 (46.1) | 58,471 (47.5) | 271,412 (45.8) | < 0.001 |
| Number of vaccinations in previous years (n, %)  0  1  2  3  4  5 | 42,364 (5.9)  47,526 (6.6)  55,189 (7.7)  61,971 (8.7)  96,326 (13.5)  412,669 (57.6) | 13,845 (11.2)  14,919 (12.1)  15,718 (12.8)  17,405 (14.1)  21,876 (17.8)  39,311 (31.9) | 28,519 (4.8)  32,607 (5.5)  39,471 (6.7)  44,566 (7.5)  74,450 (12.6)  373,358 (63.0) | < 0.001 |
| Vaccinated in season/year (n, %)  2012/13  2011/12  2010/11  2009/10  2008/09 | 631,921 (88.3)  597,992 (83.5)  554,008 (77.4)  531,614 (74.2)  476,931 (66.6) | 94,454 (76.7)  85,207 (69.2)  75,936 (61.7)  73,241 (59.5)  53,791 (43.7) | 537,467 (90.6)  512,785 (86.5)  478,072 (80.6)  458,373 (77.3)  478,072 (80.6) | < 0.001  < 0.001  < 0.001  < 0.001  < 0.001 |
| Total number of outcomes in year 2013/2014^2^ (n, %)  ILI  ARI | 782 (0.1)  9,463 (1.3) | 202 (0.2)  2,529 (2.1) | 580 (0.1)  6,934 (1.2) | < 0.001  < 0.001 |
| Hip fractures (n, %) | 668 (0.09) | 14 (0.01) | 654 (0.11) | < 0.001 |
| Urinary tract infections (n, %) | 5,911 (0.8) | 639 (0.5) | 5,272 (0.9) | < 0.001 |
| Charlson Comorbidity Index (n, %)^3^  0  1 - 2  3 - 4  > 5 | 265,367 (37.1)  330,163 (46.1)  97,928 (13.7)  22,587 (3.2) | 0 (0.0)  103,636 (84.2)  17,495 (14.2)  1,943 (1.6) | 265,367 (44.8)  226,527 (38.2)  80,433 (13.6)  20,644 (3.5) | < 0.001 |
| Index of multiple deprivation  1 - 2  3 – 4  5 | 258,352 (36.1)  299,111 (41.8)  158,582 (22.1) | 40,385 (32.8)  50,984 (41.4)  31,705 (25.8) | 217,967 (36.8)  248,127 (41.8)  126,877 (21.4) | < 0.001 |

# **Table S9.** Characteristics of individuals included in the study for the 2014/15 winter influenza season

| Characteristics | **Overall** | **< 65 years** | **≥ 65 years** | p-value^1^ |
| --- | --- | --- | --- | --- |
| N | 619,349 | 110,835 (17.9) | 508,514 (82.1) |  |
| Age (median, SD) | 75 (14.3) | 54 (13.2) | 77 (8.0) | < 0.001 |
| Male (n, %) | 285,336 (46.1) | 52,075 (47.0) | 233,261 (45.9) | < 0.001 |
| Number of vaccinations in previous years (n, %)  0  1  2  3  4  5 | 33,296 (5.4)  38,077 (6.1)  42,762 (6.9)  52,562 (8.5)  77,858 (12.6)  374,794 (60.5) | 11,270 (10.2)  12,452 (11.2)  13,053 (11.8)  14,211 (12.8)  18,165 (16.4)  41,684 (37.6) | 22,026 (4.3)  25,625 (5.0)  29,709 (5.8)  38,351 (7.5)  59,693 (11.7)  333,110 (65.5) | < 0.001 |
| Vaccinated in season/year (n, %)  2013/14  2012/13  2011/12  2010/11  2009/10 | 551,235 (89.0)  522,445 (84.4)  495,238 (80.0)  458,126 (74.0)  439,645 (71.0) | 86,460 (78.0)  78,573 (70.9)  71,537 (64.5)  63,885 (57.6)  61,816 (55.8) | 464,775 (91.4)  443,872 (87.3)  423,701 (83.3)  394,241 (77.5)  377,829 (74.3) | < 0.001  < 0.001  < 0.001  < 0.001  < 0.001 |
| Total number of outcomes in year 2014/2015^2^ (n, %)  ILI  ARI | 1,704 (0.3)  13,401 (2.2) | 423 (0.4)  3,506 (3.2) | 1,281 (0.3)  9,895 (1.9) | < 0.001  < 0.001 |
| Hip fractures (n, %) | 792 (0.13) | 15 (0.01) | 777 (0.15) | < 0.001 |
| Urinary tract infections (n, %) | 6,073 (1.0) | 673 (0.6) | 5,400 (1.06) | < 0.001 |
| Charlson Comorbidity Index (n, %)^3^  0  1 - 2  3 - 4  > 5 | 231,534 (37.4)  284,836 (46.0)  83,880 (13.5)  19,099 (3.1) | 0 (0.0)  92,690 (83.6)  16,310 (14.7)  1,835 (1.7) | 231,534 (45.5)  192,146 (37.8)  67,570 (13.3)  17,264 (3.4) | < 0.001 |
| Index of multiple deprivation  1 - 2  3 – 4  5 | 224,238 (36.2)  257,041 (41.5)  138,070 (22.3) | 36,562 (33.0)  45,701 (41.2)  28,572 (25.8) | 187,676 (36.9)  211,340 (41.6)  109,498 (21.5) | < 0.001 |

# **Table S10.** Characteristics of individuals included in the study for the 2015/16 winter influenza season

| Characteristics | **Overall** | **< 65 years** | **≥ 65 years** | p-value^1^ |
| --- | --- | --- | --- | --- |
| N | 476,616 | 87,280 (18.3) | 389,336 (81.7) |  |
| Age (median, SD) | 74 (13.9) | 54 (12.9) | 76 (7.8) | < 0.001 |
| Male (n, %) | 219,605 (46.1) | 40,816 (46.8) | 178,789 (45.9) | < 0.001 |
| Number of vaccinations in previous years (n, %)  0  1  2  3  4  5 | 23,621 (5.0)  26,534 (5.6)  30,718 (6.4)  36,905 (7.7)  60,767 (12.7)  298,071 (62.5) | 8,486 (9.7)  9,080 (10.4)  9,831 (11.3)  10,767 (12.3)  14,219 (16.3)  34,897 (40.0) | 15,135 (3.9)  17,454 (4.5)  20,887 (5.4)  26,138 (6.7)  46,548 (12.0)  263,174 (67.6) | < 0.001 |
| Vaccinated in season/year (n, %)  2014/15  2013/14  2012/13  2011/12  2010/11 | 427,560 (89.7)  409,010 (85.8)  387,985 (81.4)  367,540 (77.1)  340,013 (71.3) | 69,085 (79.2)  63,920 (73.2)  58,386 (66.9)  53,317 (61.1)  47,696 (54.6) | 358,475 (92.1)  345,090 (88.6)  329,599 (84.7)  314,223 (80.7)  292,317 (75.1) | < 0.001  < 0.001  < 0.001  < 0.001  < 0.001 |
| Total number of outcomes in the 2015/16 season (n, %)  ILI  ARI | 931 (0.2)  7,807 (1.6) | 301 (0.3)  2,211 (2.5) | 630 (0.2)  5,596 (1.4) | < 0.001  < 0.001 |
| Hip fractures (n, %) | 558 (0.12) | 14 (0.02) | 544 (0.14) | < 0.001 |
| Urinary tract infections (n, %) | 4,386 (0.9) | 524 (0.6) | 3,862 (0.0) | < 0.001 |
| Charlson Comorbidity Index (n, %)^3^  0  1 - 2  3 - 4  > 5 | 180,176 (37.8)  216,974 (45.5)  65,146 (13.7)  14,320 (3.0) | 0 (0.0)  72,082 (82.6)  13,705 (15.7)  1,493 (1.7) | 180,176 (46.3)  144,892 (37.2)  51,441 (13.2)  12,827 (3.3) | < 0.001 |
| Index of multiple deprivation  1 - 2  3 – 4  5 | 169,171 (35.5)  194,448 (40.8)  112,997 (23.7) | 27,906 (32.0)  35,898 (41.1)  23,476 (26.9) | 141,265 (36.3)  158,550 (40.7)  89,521 (23.0) | < 0.001 |

# **Table S11.** Relative influenza vaccine effectiveness of seasonal influenza vaccines against general practitioner-diagnosed influenza-like illness (GP-ILI) and medically attended acute respiratory illness (MAARI) during influenza seasons among individuals vaccinated in both the current and prior influenza season.

| **Season** | **< 65 years** | | | | **≥ 65 years** | | | |
| --- | --- | --- | --- | --- | --- | --- | --- | --- |
|  | **Outcome (n, %)^1^** | **n (%)^2^** | **HR^4^** | **95% CI** | **Outcome (n, %)^2^** | **n (%)^1^** | **HR^4^** | **95% CI** |
| ***General practitioner-diagnosed influenza-like illness (GP-ILI)*** | | | | | | | | |
| 2011/12 | **358 (0.3) ***** |  |  | | **1,109 (0.2) ***** |  |  | |
| Both seasons^2^ | 266 (0.3) | 93,972 (73.6) | 1.01 | 0.80, 1.30 | 990 (0.2) | 612,379 (88.1) | 1.04 | 0.86, 1.24 |
| 2012/13 | **617 (0.5) ***** |  |  | | **1,584 (0.2) ***** |  |  | |
| Both seasons | 487 (0.5) | 98,654 (75.7) | 1.12 | 0.93, 1.38 | 1,439 (0.2) | 598,986 (90.4) | 0.96 | 0.83, 1.16 |
| 2013/14 | **202 (0.2) ***** |  |  | | **580 (0.1) ***** |  |  | |
| Both seasons | 164 (0.2) | 94,457 (76.6) | 1.25 | 0.88, 1.86 | 530 (0.1) | 537,467 (90.6) | 1.02 | 0.77, 1.39 |
| 2014/15 | **423 (0.4) ***** |  |  | | **1,281 (0.3) ***** |  |  | |
| Both seasons | 343 (0.4) | 86,460 (78.0) | 1.13 | 0.90, 1.48 | 1,181 (0.3) | 464,775 (91.4) | 1.00 | 0.81, 1.24 |
| 2015/16 | **301 (0.4) ***** |  |  | | **630 (0.2) ***** |  |  | |
| Both seasons | 241 (0.4) | 69,085 (79.2) | 1.04 | 0.78, 1.39 | 574 (0.2) | 358,475 (92.1) | 0.82 | 0.64, 1.11 |
| ***Medically attended acute respiratory illness (MAARI)*** | | | | | | | | |
| 2011/12 | **3,672 (2.9) ***** |  |  | | **11,587 (1.7) ***** |  |  | |
| Both seasons | 2,753 (3.0) | *saa.*^5^ | 1.05 | 0.98, 1.14 | 10,356 (1.7) | *saa.* | 1.08 | 1.02, 1.15 |
| 2012/13 | **4,679 (3.7) ***** |  |  | | **12,911 (2.0) ***** |  |  | |
| Both seasons | 3,691 (3.7) |  | 1.15 | 1.07, 1.24 | 11,830 (2.0) |  | 1.08 | 1.02. 1.16 |
| 2013/14 | **2,529 (2.1) ***** |  |  | | **6,934 (1.2) ***** |  |  | |
| Both seasons | 2,012 (2.1) |  | 1.13 | 1.03, 1.25 | 6,330 (1.2) |  | 1.02 | 0.94, 1.11 |
| 2014/15 | **3,506 (3.2) ***** |  |  | | **9,895 (2.0) ***** |  |  | |
| Both seasons | 2,859 (3.3) |  | 1.17 | 1.08, 1.29 | 9,212 (2.0) |  | 1.15 | 1.06, 1.25 |
| 2015/16 | **2,211 (2.5) ***** |  |  | | **5,596 (1.4) ***** |  |  | |
| Both seasons | 1,827 (2.6) |  | 1.22 | 1.10, 1.37 | 5,191 (1.5) |  | 1.03 | 0.94, 1.14 |

^1^ Percentages of outcomes **(in bold)** were calculated from the total of individuals in each age group; p-value for chi-square tests comparing ILI/ARI cases for both groups are indicated as the following: * p<0.05, ** p<0.01, *** p<0.001. Percentages of outcomes were also calculated for the group that is vaccinated in both seasons.

^2^ Percentage was calculated from the total of individuals in each age group stratified by vaccination status

^3^ Both seasons = vaccinated in current and prior influenza seasons

^4^ Hazard ratios are reported referent to individuals who have received influenza vaccination for the current influenza season only. All models adjusted for sex, Charlson comorbidity index and index of multiple deprivation

^5^ *saa.* = Same as above

# **Table S12**. Relative influenza vaccine effectiveness of seasonal influenza vaccines against general practitioner-diagnosed influenza-like illness (GP-ILI) and medically attended acute respiratory illness (MAARI) during influenza seasons by the number of influenza vaccinations received in the previous 5 seasons

| **Season** | **Outcome (n, %)^1^** | **n (%)^2^** | **< 65 years** | | **Outcome (n, %)** | **n (%)** | **≥ 65 years** | |  |  |
| --- | --- | --- | --- | --- | --- | --- | --- | --- | --- | --- |
|  |  |  | HR^3^ | 95% CI |  |  | HR | 95% CI |  |  |
| ***General practitioner-diagnosed influenza-like illness (GP-ILI)*** | | | | | | | | | |  |
| 2011/12 |  |  |  | |  |  |  | |  |  |
| Current + 1 prior  Current + 2 prior  Current + 3 prior  Current + 4 prior  Current + 5 prior | 54 (0.27)  58 (0.26)  51 (0.30)  53 (0.30)  96 (0.29) | 20,092 (15.7)  21,925 (17.2)  17,284 (13.5)  17,734 (13.9)  33,063 (25.9) | 1.03  1.00  1.11  1.09  1.07 | 0.69, 1.53  0.68, 1.48  0.72, 1.69  0.74, 1.69  0.74, 1.55 | 67 (0.15)  88 (0.17)  91 (0.16)  142 (0.17)  662 (0.16) | 45,755 (6.7)  53,096 (7.6)  57,283 (8.2)  83,818 (12.1)  410,733 (59.1) | 1.06  1.16  1.10  1.14  1.05 | 0.75, 1.52  0.84, 1.61  0.78, 1.52  0.86, 1.54  0.82, 1.37 |  |  |
| 2012/13 |  |  |  | |  |  |  | |  | |
| Current + 1 prior  Current + 2 prior  Current + 3 prior  Current + 4 prior  Current + 5 prior | 71 (0.40)  93 (0.49)  95 (0.45)  91 (0.46)  207 (0.56) | 17,606 (13.5)  18,812 (14.4)  20,975 (16.1)  19,732 (15.1)  37,227(28.6) | 1.04  1.27  1.13  1.14  1.35 | 0.73, 1.51  0.92, 1.79  0.84, 1.58  0.83, 1.64  1.00, 1.88 | 85 (0.20)  94 (0.21)  128 (0.23)  211 (0.26)  991 (0.25) | 42,409 (6.4)  44,616 (6.7)  55,830 (8.4)  82,579 (12.5)  402,980 (60.8) | 0.89  0.90  0.97  1.06  0.99 | 0.65, 1.21  0.67, 1.23  0.73, 1.23  0.83, 1.42  0.79, 1.28 |  |  |
| 2013/14 |  |  |  | |  |  |  | |  | |
| Current + 1 prior  Current + 2 prior  Current + 3 prior  Current + 4 prior  Current + 5 prior | 27 (0.18)  22 (0.14)  31 (0.18)  36 (0.16)  68 (0.17) | 14,919 (12.1)  15,718 (12.8)  17,405 (14.1)  21,876 (17.8)  39,311 (31.9) | 1.37  1.03  1.32  1.20  1.25 | 0.74, 2.71  0.54, 2.17  0.76, 2.55  0.68, 2.35  0.74, 2.36 | 28 (0.09)  24 (0.06)  38 (0.09)  73 (0.10)  396 (0.11) | 32,607 (5.5)  39,471 (6.7)  44,566 (7.5)  74,450 (12.6)  373,358 (63.0) | 1.15  0.80  1.10  1.25  1.32 | 0.66, 2.16  0.45, 1.46  0.63, 1.99  0.78, 2.25  0.87, 2.25 |  |  |
| 2014/15 |  |  |  | |  |  |  | |  |  |
| Current + 1 prior  Current + 2 prior  Current + 3 prior  Current + 4 prior  Current + 5 prior | 43 (0.35)  46 (0.35)  62 (0.44)  79 (0.43)  169 (0.41) | 12,452 (11.2)  13,053 (11.8)  14,211 (12.8)  18,165 (16.4)  41,684 (37.6) | 1.57  1.57  1.96  1.92  1.77 | 0.96, 2.67  1.01, 2.74  1.27, 3.26  1.21, 3.12  1.17, 2.90 | 51 (0.20)  74 (0.25)  68 (0.18)  154 (0.26)  885 (0.27) | 25,625 (5.0)  29,709 (5.8)  38,351 (7.5)  59,693 (11.7)  333,110 (65.5) | 0.85  1.05  0.72  1.03  1.01 | 0.55, 1.24  0.72, 1.51  0.51, 1.02  0.76, 1.44  0.76, 1.39 |  |  |
| 2015/16 |  |  |  | |  |  |  | |  |  |
| Current + 1 prior  Current + 2 prior  Current + 3 prior  Current + 4 prior  Current + 5 prior | 30 (0.33)  38 (0.39)  30 (0.28)  51 (0.36)  126 (0.36) | 9,080 (10.4)  9,831 (11.3)  10,767 (12.3)  14,219 (16.3)  34,897 (40.0) | 1.07  1.24  0.87  1.13  1.13 | 0.62, 1.83  0.74, 2.13  0.50, 1.55  0.70, 1.89  0.79, 1.80 | 19 (0.11)  36 (0.17)  36 (0.14)  95 (0.20)  425 (0.16) | 17,454 (4.5)  20,887 (5.4)  26,138 (6.7)  46,548 (12.0)  263,174 (67.6) | 0.85  1.32  1.04  1.48  1.15 | 0.44, 1.62  0.76, 2.45  0.59, 1.95  0.94, 2.73  0.75, 2.17 |  |  |
| ***Medically attended acute respiratory illness (MAARI)*** | | | | | | | | | |  |
| 2011/12 |  |  |  | |  |  |  | |  |  |
| Current + 1 prior  Current + 2 prior  Current + 3 prior  Current + 4 prior  Current + 5 prior | 563 (2.80)  581 (2.65)  501 (2.90)  561 (3.16)  999 (3.02) | *saa.*^4^ | 1.05  0.99  1.07  1.15  1.10 | 0.92, 1.17  0.87, 1.12  0.94, 1.22  1.02, 1.30  0.98, 1.22 | 746 (1.63)  841 (1.58)  1,000 (1.75)  1,419 (1.69)  6,951 (1.69) | *saa.* | 1.11  1.06  1.17  1.11  1.10 | 1.00, 1.23  0.96, 1.19  1.06, 1.29  1.02, 1.22  1.01, 1.19 |  |  |
| 2012/13 |  |  |  | |  |  |  | |  |  |
| Current + 1 prior  Current + 2 prior  Current + 3 prior  Current + 4 prior  Current + 5 prior | 610 (3.46)  662 (3.52)  746 (3.56)  675 (3.42)  1,507 (4.05) |  | 1.13  1.14  1.14  1.08  1.27 | 1.00, 1.26  1.01, 1.28  1.02, 1.28  0.96, 1.21  1.14, 1.41 | 691 (1.63)  778 (1.74)  1,071 (1.92)  1,626 (1.97)  8,187 (2.03) |  | 0.97  1.02  1.10  1.11  1.12 | 0.87, 1.09  0.91, 1.13  0.99, 1.21  1.01, 1.22  1.03, 1.22 |  |  |
| 2013/14 |  |  |  | |  |  |  | |  |  |
| Current + 1 prior  Current + 2 prior  Current + 3 prior  Current + 4 prior  Current + 5 prior | 253 (1.70)  305 (1.94)  372 (2.14)  454 (2.08)  911 (2.32) |  | 0.97  1.11  1.20  1.17  1.28 | 0.82, 1.16  0.93, 1.31  1.03, 1.41  1.01, 1.36  1.12, 1.48 | 346 (1.06)  422 (1.07)  507 (1.14)  913 (1.23)  4,463 (1.20) |  | 1.04  1.04  1.08  1.14  1.10 | 0.89, 1.21  0.88, 1.21  0.93, 1.26  1.00, 1.33  0.97, 1.24 |  |  |
| 2014/15 |  |  |  | |  |  |  | |  |  |
| Current + 1 prior  Current + 2 prior  Current + 3 prior  Current + 4 prior  Current + 5 prior | 357 (2.87)  362 (2.77)  445 (3.13)  614 (3.38)  1,476 (3.54) |  | 1.23  1.18  1.31  1.41  1.45 | 1.06, 1.44  0.99, 1.39  1.13, 1.54  1.22, 1.63  1.27, 1.67 | 421 (1.64)  494 (1.66)  611 (1.59)  1,172 (1.96)  6,872 (2.06) |  | 1.06  1.05  0.99  1.20  1.21 | 0.92, 1.23  0.91, 1.22  0.87, 1.13  1.07, 1.35  1.10, 1.36 |  |  |
| 2015/16 |  |  |  | |  |  |  | |  |  |
| Current + 1 prior  Current + 2 prior  Current + 3 prior  Current + 4 prior  Current + 5 prior | 217 (2.39)  250 (2.54)  267 (2.48)  356 (2.50)  963 (2.76) |  | 1.25  1.33  1.28  1.30  1.42 | 1.02, 1.53  1.10, 1.65  1.07, 1.58  1.08, 1.60  1.22, 1.70 | 204 (1.17)  281 (1.35)  365 (1.40)  670 (1.44)  3,910 (1.49) |  | 1.03  1.16  1.19  1.20  1.21 | 0.83, 1.27  0.97, 1.42  0.99, 1.45  1.02, 1.43  1.04, 1.43 |  |  |

^1^  Percentages of outcomes were calculated from the number of individuals in each group

^2^ Percentages of number of individuals in each group were calculated from the total number of individuals in each age group

^3^ Hazard ratios are reported referent to individuals who received influenza vaccination for the current influenza season only. All models adjusted for sex, Charlson comorbidity index and index of multiple deprivation

^4^ *saa.* = Same as above

# **Table S13.** Relative influenza vaccine effectiveness of seasonal influenza vaccines against medically attended acute respiratory illness (MAARI) outside influenza seasons among individuals vaccinated in both the current and prior influenza season

| **Season** | **< 65 years** | | | | | **≥ 65 years** | | | |
| --- | --- | --- | --- | --- | --- | --- | --- | --- | --- |
|  | **Outcome (n, %)^1^** | **n (%)^2^** | | **HR^4^** | **95% CI** | **Outcome (n, %)^2^** | **n (%)^1^** | **HR^4^** | **95% CI** |
| 2011/12 | **1,506 (1.20) ***** | |  | | | **4,533 (0.67) ***** |  |  | |
| Both seasons | 1,129 (1.22) | 92,691 (73.7) | | 1.08 | 0.95, 1.21 | 4,062 (0.68) | 597,758 (88.1) | 1.12 | 1.02, 1.24 |
| 2012/13 | **979 (0.76) ***** | |  | | | **2,793 (0.43) ***** |  |  | |
| Both seasons | 769 (0.79) | 97,203 (75.7) | | 1.17 | 1.00, 1.38 | 2,525 (0.43) | 582,597 (90.4) | 0.96 | 0.85, 1.09 |
| 2013/14 | **1,068 (0.88) ***** | |  | | | **2,905 (0.50) ***** |  |  | |
| Both seasons | 818 (0.88) | 93,241 (76.7) | | 0.99 | 0.87, 1.15 | 2,653 (0.50) | 527,016 (90.6) | 1.05 | 0.93, 1.20 |
| 2014/15 | **794 (0.73) ***** | |  | | | **2,178 (0.44) ***** |  |  | |
| Both seasons | 631 (0.74) | 85,404 (78.0) | | 1.10 | 0.93, 1.35 | 2,027 (0.45) | 453,395 (91.4) | 1.22 | 1.05, 1.48 |
| 2015/16 | **579 (0.67) ***** | |  | | | **1,367 (0.36) ***** |  |  | |
| Both seasons | 462 (0.68) | 68,306 (79.2) | | 1.04 | 0.86, 1.25 | 1,256 (0.36) | 350,568 (92.1) | 0.91 | 0.76, 1.14 |

^1^ Percentages of outcomes **(in bold)** were calculated from the total of individuals in each age group; p-value for chi-square tests comparing ARI cases for both groups are indicated as the following: * p<0.05, ** p<0.01, *** p<0.001. Percentages of outcomes were also calculated for the group that is vaccinated in both seasons.

^2^ Percentage was calculated from the total of individuals in each age group stratified by vaccination status

^3^ Both seasons = vaccinated in current and prior influenza seasons

^4^ Hazard ratios are reported referent to individuals who have received influenza vaccination for the current influenza season only. All models adjusted for sex, Charlson comorbidity index and index of multiple deprivation

^5^ This analysis is not conducted for influenza-like illness (ILI) due to the low number of cases

# **Table S14**. Relative influenza vaccine effectiveness of seasonal influenza vaccines against medically attended acute respiratory illness (MAARI) outside influenza seasons by the number of influenza vaccinations received in the previous 5 seasons

| **Season** | **Outcome (n, %)^1^** | **n (%)^2^** | **< 65 years** | | **Outcome (n, %)** | **n (%)** | **≥ 65 years** | |
| --- | --- | --- | --- | --- | --- | --- | --- | --- |
|  |  |  | HR^3^ | 95% CI |  |  | HR | 95% CI |
| 2011/12 |  |  |  | |  |  |  | |
| Current + 1 prior  Current + 2 prior  Current + 3 prior  Current + 4 prior  Current + 5 prior | 230 (1.17)  284 (1.31)  191 (1.12)  228 (1.30)  380 (1.16) | 19,709 (15.7)  21,603 (17.2)  17,040 (13.5)  17,517 (13.9)  32,692 (26.0) | 1.05  1.19  1.01  1.17  1.05 | 0.87, 1.27  0.98, 1.41  0.83, 1.24  0.97, 1.41  0.88, 1.24 | 295 (0.66)  340 (0.65)  390 (0.70)  549 (0.67)  2,728 (0.68) | 44,743 (6.6)  51,916 (7.7)  55,915 (8.2)  81,502 (12.0)  400,731 (59.1) | 1.20  1.19  1.26  1.20  1.20 | 1.02, 1.45  1.01, 1.41  1.07, 1.48  1.03, 1.41  1.05, 1.38 |
| 2012/13 |  |  |  | |  |  |  | |
| Current + 1 prior  Current + 2 prior  Current + 3 prior  Current + 4 prior  Current + 5 prior | 116 (0.67)  172 (0.93)  159 (0.77)  139 (0.72)  287 (0.78) | 17,317 (13.5)  18,511 (14.4)  20,647 (16.1)  19,434 (15.1)  36,770 (28.7) | 0.99  1.37  1.13  1.05  1.15 | 0.76, 1.30  1.07, 1.74  0.89, 1.48  0.81, 1.36  0.94, 1.44 | 148 (0.36)  220 (0.51)  246 (0.45)  357 (0.45)  1,693 (0.43) | 41,687 (6.5)  43,555 (6.8)  54,341 (8.4)  79,999 (12.4)  391,358 (60.7) | 0.92  1.27  1.12  1.09  1.04 | 0.72, 1.16  1.02, 1.59  0.93, 1.41  0.90, 1.36  0.88, 1.26 |
| 2013/14 |  |  |  | |  |  |  | |
| Current + 1 prior  Current + 2 prior  Current + 3 prior  Current + 4 prior  Current + 5 prior | 124 (0.84)  132 (0.85)  163 (0.95)  210 (0.97)  327 (0.84) | 14,723 (12.1)  15,522 (12.8)  17,215 (14.2)  21,639 (17.8)  38,904 (32.0) | 1.03  1.04  1.16  1.19  1.03 | 0.81, 1.32  0.81, 1.33  0.94, 1.47  0.95, 1.48  0.84, 1.29 | 139 (0.43)  170 (0.44)  208 (0.48)  390 (0.54)  1,873 (0.51) | 32,187 (5.5)  38,919 (6.7)  43,715 (7.5)  72,758 (12.5)  365,673 (62.9) | 0.96  0.96  1.03  1.14  1.08 | 0.76, 1.24  0.78, 1.22  0.83, 1.28  0.94, 1.41  0.92, 1.31 |
| 2014/15 |  |  |  | |  |  |  | |
| Current + 1 prior  Current + 2 prior  Current + 3 prior  Current + 4 prior  Current + 5 prior | 91 (0.74)  88 (0.68)  92 (0.66)  135 (0.75)  320 (0.78) | 12,288 (11.2)  12,875 (11.8)  14,034 (12.8)  17,935 (16.4)  41,199 (37.7) | 1.22  1.12  1.08  1.23  1.28 | 0.90, 1.74  0.80, 1.53  0.79, 1.49  0.95, 1.65  1.00, 1.71 | 96 (0.38)  119 (0.41)  150 (0.40)  246 (0.42)  195 (0.46) | 25,261 (5.1)  29,216 (5.9)  37,591 (7.6)  58,002 (11.7)  324,334 (65.4) | 1.13  1.21  1.17  1.23  1.32 | 0.85, 1.54  0.92, 1.61  0.90, 1.54  0.95, 1.60  1.05, 1.69 |
| 2015/16 |  |  |  | |  |  |  | |
| Current + 1 prior  Current + 2 prior  Current + 3 prior  Current + 4 prior  Current + 5 prior | 69 (0.77)  65 (0.67)  68 (0.64)  107 (0.76)  226 (0.65) | 8,967 (10.4)  9,701 (11.3)  10,634 (12.3)  14,064 (16.3)  34,523 (40.0) | 1.47  1.28  1.21  1.43  1.24 | 1.01, 2.19  0.88, 1.90  0.82, 1.79  1.01, 2.10  0.91, 1.76 | 53 (0.31)  59 (0.29)  88 (0.34)  172 (0.38)  935 (0.36) | 17,173 (4.5)  20,574 (5.4)  25,659 (6.7)  45,419 (11.9)  256,964 (67.5) | 0.75  0.68  0.80  0.85  0.79 | 0.52, 1.08  0.47, 0.96  0.59, 1.15  0.65, 1.19  0.63, 1.07 |

^1^  Percentages of outcomes were calculated from the number of individuals in each group

^2^ Percentages of number of individuals in each group were calculated from the total number of individuals in each age group

^3^ Hazard ratios are reported referent to individuals who received influenza vaccination for the current influenza season only. All models adjusted for sex, Charlson comorbidity index and index of multiple deprivation

^4^ This analysis is not conducted for influenza-like illness (ILI) due to the low number of cases

# **Table S15.** Relative influenza vaccine effectiveness of seasonal influenza vaccines against hip fractures and urinary tract infections during influenza seasons among individuals vaccinated in both the current and prior influenza season

| **Season** | **< 65 years** | | | | **≥ 65 years** | | | |
| --- | --- | --- | --- | --- | --- | --- | --- | --- |
|  | **Outcome (n, %)^1^** | **n (%)^2^** | **HR^4^** | **95% CI** | **Outcome (n, %)^2^** | **n (%)^1^** | **HR^4^** | **95% CI** |
| ***Hip fractures*** | | | | | | | | |
| 2011/12 | **13 (0.01) ***** |  |  | | **897 (0.13) ***** |  |  | |
| Both seasons^2^ | 10 (0.01) | 93,972 (73.6) | - | - | 800 (0.13) | 612,379 (88.1) | 0.98 | 0.81, 1.25 |
| 2012/13 | **18 (0.01) ***** |  |  | | **1,103 (0.17) ***** |  |  | |
| Both seasons | 14 (0.01) | 98,654 (75.7) | - | - | 1,008 (0.17) | 598,986 (90.4) | 0.98 | 0.80, 1.22 |
| 2013/14 | **14 (0.01) ***** |  |  | | **654 (0.11) ***** |  |  | |
| Both seasons | 11 (0.01) | 94,454 (76.8) | - | - | 597 (0.11) | 537,467 (90.6) | 0.94 | 0.70, 1.24 |
| 2014/15 | **15 (0.01) ***** |  |  | | **777 (0.15) ***** |  |  | |
| Both seasons | 11 (0.01) | 86,460 (78.0) | - | - | 719 (0.15) | 464,775 (91.4) | 1.01 | 0.78, 1.36 |
| 2015/16 | **14 (0.02) ***** |  |  | | **544 (0.14) ***** |  |  | |
| Both seasons | 10 (0.01) | 69,085 (79.2) | - | - | 508 (0.14) | 358,475 (92.1) | 1.06 | 0.78, 1.56 |
| ***Urinary tract infections*** | | | | | | | | |
| 2011/12 | **686 (0.54) ***** |  |  | | **7,525 (1.08) ***** |  |  | |
| Both seasons | 532 (0.57) | *saa.*^6^ | 1.17 | 0.99, 1.41 | 6,868 (1.12) | *saa.*^6^ | 1.26 | 1.17, 1.36 |
| 2012/13 | **899 (0.69) ***** |  |  | | **8,226 (1.24) ***** |  |  | |
| Both seasons | 717 (0.73) |  | 1.18 | 1.00, 1.40 | 7,653 (1.28) |  | 1.25 | 1.15, 1.36 |
| 2013/14 | **639 (0.52) ***** |  |  | | **5,272 (0.89) ***** |  |  | |
| Both seasons | 518 (0.55) |  | 1.20 | 0.99, 1.50 | 4,877 (0.91) |  | 1.13 | 1.02, 1.26 |
| 2014/15 | **673 (0.61) ***** |  |  | | **5,400 (1.06) ***** |  |  | |
| Both seasons | 546 (0.63) |  | 1.13 | 0.94, 1.37 | 5,036 (1.08) |  | 1.14 | 1.02, 1.27 |
| 2015/16 | **524 (0.60) ***** |  |  | | **3,862 (0.99) ***** |  |  | |
| Both seasons | 416 (0.60) |  | 0.93 | 0.76, 1.16 | 3,617 (1.01) |  | 1.12 | 0.99, 1.29 |

Note:

^1^ Percentages of outcomes **(in bold)** were calculated from the total of individuals in each age group; p-value for chi-square tests comparing ILI/ARI cases for both groups are indicated as the following: * p<0.05, ** p<0.01, *** p<0.001. Percentages of outcomes were also calculated for the group that is vaccinated in both seasons.

^2^ Percentage was calculated from the total of individuals in each age group stratified by vaccination status

^3^ Both seasons = vaccinated in current and prior influenza seasons

^4^ Hazard ratios are reported referent to individuals who have received influenza vaccination for the current influenza season only. All models adjusted for sex, Charlson comorbidity index and index of multiple deprivation

^5^ This analysis was not conducted for hip fractures in the below 65 years old group because of the low number of cases.

^6^ *saa.* = Same as above

**Table S16.** Relative influenza vaccine effectiveness of seasonal influenza vaccines against hip fractures and urinary tract infections during influenza seasons by the number of influenza vaccinations received in the previous 5 seasons

| **Season** | **Outcome (n, %)^1^** | **n (%)^2^** | **< 65 years** | | **Outcome (n, %)** | **n (%)** | **≥ 65 years** | |  |
| --- | --- | --- | --- | --- | --- | --- | --- | --- | --- |
|  |  |  | HR^3^ | 95% CI |  |  | HR | 95% CI |  |
| ***Hip fractures*** | | | | | | | | | |
| 2011/12 |  |  |  | |  |  |  | |  |
| Current + 1 prior  Current + 2 prior  Current + 3 prior  Current + 4 prior  Current + 5 prior | 3 (0.01)  2 (0.01)  3 (0.02)  1 (0.01)  2 (0.01) | 20,092 (15.7)  21,925 (17.2)  17,284 (13.5)  17,734 (13.9)  33,063 (25.9) | - | - | 34 (0.07)  39 (0.07)  64 (0.11)  120 (0.14)  620 (0.15) | 45,755 (6.6)  53,096 (7.6)  57,283 (8.2)  83,818 (12.1)  410,733 (59.1) | 1.56  1.48  2.21  2.65  2.74 | 0.88, 2.76  0.88, 2.80  1.39, 3.83  1.72, 4.60  1.85, 4.59 |  |
| 2012/13 |  |  |  | |  |  |  | |  |
| Current + 1 prior  Current + 2 prior  Current + 3 prior  Current + 4 prior  Current + 5 prior | 1 (0.01)  3 (0.02)  3 (0.01)  4 (0.02)  6 (0.02) | 17,606 (13.5)  18,812 (14.4)  20,975 (16.1)  19,732 (15.1)  37,227 (28.6) | - | - | 37 (0.09)  36 (0.08)  63 (0.11)  146 (0.18)  788 (0.20) | 42,409 (6.4)  44,616 (6.7)  55,830 (8.4)  82,579 (12.5)  402,980 (60.8) | 0.87  0.75  1.01  1.53  1.62 | 0.53, 1.40  0.46, 1.25  0.67, 1.61  1.09, 2.40  1.18, 2.47 |  |
| 2013/14 |  |  |  | |  |  |  | |  |
| Current + 1 prior  Current + 2 prior  Current + 3 prior  Current + 4 prior  Current + 5 prior | 2 (0.01)  2 (0.01)  6 (0.03)  1 (0.00)  2 (0.01) | 14,919 (12.1)  15,718 (12.8)  17,405 (14.1)  21,876 (17.8)  39,311 (31.9) | - | - | 13 (0.04)  17 (0.04)  40 (0.09)  88 (0.12)  477 (0.13) | 32,607 (5.5)  39,471 (6.7)  44,566 (7.5)  74,450 (12.6)  373,358 (63.0) | 0.57  0.58  1.16  1.45  1.48 | 0.24, 1.15  0.30, 1.13  0.69, 2.06  0.89, 2.43  0.95, 2.58 |  |
| 2014/15 |  |  |  | |  |  |  | |  |
| Current + 1 prior  Current + 2 prior  Current + 3 prior  Current + 4 prior  Current + 5 prior | 3 (0.02)  2 (0.02)  2 (0.01)  0 (0.00)  6 (0.01) | 12,452 (11.2)  13,053 (11.8)  14,211 (12.8)  18,165 (16.4)  41,684 (37.6) | - | - | 21 (0.08)  23 (0.08)  34 (0.09)  90 (0.15)  594 (0.18) | 25,625 (5.0)  29,709 (5.8)  38,351 (7.5)  59,693 (11.7)  333,110 (65.5) | 1.15  1.06  1.16  1.87  2.08 | 0.57, 2.32  0.54, 2.10  0.64, 2.25  1.12, 3.42  1.34, 3.87 |  |
| 2015/16 |  |  |  | |  |  |  | |  |
| Current + 1 prior  Current + 2 prior  Current + 3 prior  Current + 4 prior  Current + 5 prior | 1 (0.01)  0 (0.00)  2 (0.02)  5 (0.04)  5 (0.01) | 9,080 (10.4)  9,831 (11.3)  10,767 (12.3)  14,219 (16.3)  34,897 (40.0) | - | - | 14 (0.08)  16 (0.08)  23 (0.09)  61 (0.13)  424 (0.16) | 17,454 (4.5)  20,887 (5.4)  26,138 (6.7)  46,548 (12.0)  263,174 (67.6) | 1.93  1.78  1.98  2.80  3.22 | 0.80, 6.93  0.74, 6.23  0.88, 6.35  1.38, 9.37  1.66, 9.80 |  |
| ***Urinary Tract Infections*** | | | | | | | | | |
| 2011/12 |  |  |  | |  |  |  | |  |
| Current + 1 prior  Current + 2 prior  Current + 3 prior  Current + 4 prior  Current + 5 prior | 91 (0.45)  106 (0.48)  98 (0.57)  106 (0.60)  204 (0.62) | *saa.*^5^ | 0.97  1.01  1.17  1.18  1.21 | 0.72, 1.31  0.76, 1.36  0.87, 1.55  0.91, 1.60  0.94, 1.59 | 370 (0.81)  494 (0.93)  534 (0.93)  1,002 (1.20)  4,858 (1.18) | *saa.*^5^ | 1.25  1.38  1.37  1.67  1.60 | 1.06, 1.48  1.20, 1.62  1.17, 1.60  1.45, 1.91  1.42, 1.82 |  |
| 2012/13 |  |  |  | |  |  |  | |  |
| Current + 1 prior  Current + 2 prior  Current + 3 prior  Current + 4 prior  Current + 5 prior | 111 (0.63)  111 (0.59)  149 (0.71)  150 (0.76)  288 (0.77) |  | 1.06  0.99  1.16  1.22  1.20 | 0.81, 1.41  0.75, 1.52  0.90, 1.52  0.96, 1.62  0.95, 1.52 | 335 (0.79)  420 (0.94)  570 (1.02)  1,037 (1.26)  5,661 (1.40) |  | 1.27  1.44  1.49  1.78  1.92 | 1.07, 1.52  1.22, 1.69  1.29, 1.76  1.54, 2.09  1.67, 2.21 |  |
| 2013/14 |  |  |  | |  |  |  | |  |
| Current + 1 prior  Current + 2 prior  Current + 3 prior  Current + 4 prior  Current + 5 prior | 70 (0.47)  75 (0.48)  81 (0.47)  115 (0.53)  246 (0.63) |  | 1.18  1.17  1.14  1.27  1.45 | 0.80, 1.69  0.84, 1.73  0.81, 1.57  0.92, 1.78  1.11, 2.02 | 171 (0.52)  234 (0.59)  312 (0.70)  714 (0.96)  3,694 (0.99) |  | 0.98  1.06  1.20  1.56  1.56 | 0.78, 1.19  0.87, 1.28  0.99, 1.46  1.31, 1.86  1.33, 1.85 |  |
| 2014/15 |  |  |  | |  |  |  | |  |
| Current + 1 prior  Current + 2 prior  Current + 3 prior  Current + 4 prior  Current + 5 prior | 49 (0.39)  78 (0.60)  84 (0.59)  107 (0.59)  300 (0.72) |  | 0.76  1.14  1.10  1.07  1.29 | 0.53, 1.14  0.80, 1.60  0.78, 1.58  0.80, 1.50  1.00, 1.76 | 172 (0.67)  189 (0.64)  323 (0.84)  618 (1.04)  3,980 (1.19) |  | 1.18  1.08  1.39  1.62  1.80 | 0.94, 1.50  0.88, 1.37  1.15, 1.74  1.36, 1.99  1.52, 2.21 |  |
| 2015/16 |  |  |  | |  |  |  | |  |
| Current + 1 prior  Current + 2 prior  Current + 3 prior  Current + 4 prior  Current + 5 prior | 44 (0.48)  65 (0.66)  52 (0.48)  88 (0.62)  225 (0.64) |  | 0.79  1.06  0.75  0.93  0.94 | 0.52, 1.18  0.75, 1.54  0.50, 1.08  0.68, 1.36  0.72, 1.33 | 96 (0.55)  146 (0.70)  217 (0.83)  426 (0.92)  2,891 (1.10) |  | 0.92  1.14  1.30  1.34  1.54 | 0.69, 1.23  0.86, 1.45  1.01, 1.67  1.08, 1.71  1.26, 1.94 |  |

^1^  Percentages of outcomes were calculated from the number of individuals in each group

^2^ Percentages of number of individuals in each group were calculated from the total number of individuals in each age group

^3^ Hazard ratios are reported referent to individuals who received influenza vaccination for the current influenza season only. All models adjusted for sex, Charlson comorbidity index and index of multiple deprivation

^4^ This analysis was not conducted for hip fractures in the below 65 years old group because of the low number of cases

^5^ *saa.* = Same as above.
